# Incidence of cancer in people with CKD not requiring kidney replacement therapy: a systematic review and meta-analysis

**DOI:** 10.1093/ckj/sfaf084

**Published:** 2025-03-21

**Authors:** Benjamin M P Elyan, Beatrix Tan, Emilie Lambourg, David A McAllister, Rob J Jones, Ninian N Lang, Patrick B Mark, Jennifer S Lees, Samira Bell

**Affiliations:** School of Cardiovascular and Metabolic Health, College of Medical and Veterinary Life Sciences, University of Glasgow, Glasgow, UK; Renal Medicine, Queen Elizabeth University Hospital, NHS Greater Glasgow and Clyde, 1345 Govan Road, Glasgow, UK; Renal Unit, Ninewells Hospital, Dundee, UK; Division of Population Health and Genomics, Medical Research Institute, University of Dundee, Dundee, UK; School of Health & Wellbeing, College of Medical and Veterinary Life Sciences, University of Glasgow, Glasgow, UK; Renal Medicine, Queen Elizabeth University Hospital, NHS Greater Glasgow and Clyde, 1345 Govan Road, Glasgow, UK; School of Cancer Sciences, College of Medical and Veterinary Life Sciences, University of Glasgow, Glasgow, UK; School of Cardiovascular and Metabolic Health, College of Medical and Veterinary Life Sciences, University of Glasgow, Glasgow, UK; Renal Medicine, Queen Elizabeth University Hospital, NHS Greater Glasgow and Clyde, 1345 Govan Road, Glasgow, UK; School of Cardiovascular and Metabolic Health, College of Medical and Veterinary Life Sciences, University of Glasgow, Glasgow, UK; Renal Medicine, Queen Elizabeth University Hospital, NHS Greater Glasgow and Clyde, 1345 Govan Road, Glasgow, UK; School of Cardiovascular and Metabolic Health, College of Medical and Veterinary Life Sciences, University of Glasgow, Glasgow, UK; Renal Medicine, Queen Elizabeth University Hospital, NHS Greater Glasgow and Clyde, 1345 Govan Road, Glasgow, UK; Renal Unit, Ninewells Hospital, Dundee, UK; Division of Population Health and Genomics, Medical Research Institute, University of Dundee, Dundee, UK

**Keywords:** cancer incidence, chronic kidney disease, onconephrology, systematic review

## Abstract

**Background:**

Cancer incidence in people with chronic kidney disease (CKD) who do not require kidney replacement therapy remains inadequately characterized. This systematic review aimed to establish whether there is an elevated incidence of cancer in people with CKD.

**Methods:**

A systematic search of three online bibliographic databases until 17 January 2023 identified studies reporting cancer incidence in CKD cohorts (PROSPERO CRD42022359690). Meta-analyses using inverse variance method compared incidence rates in individuals with low estimated glomerular filtration rate (eGFR) (<60 mL/min/1.73 m^2^) with available cohorts with normal eGFR (≥60 mL/min/1.73 m^2^ or both 60–89 and ≥90 mL/min/1.73 m^2^) for all cancers and site-specific cancers. Multiple meta-regression analyses explored associations of eGFR and age.

**Results:**

In 27 studies (5 519 778 people with CKD), from 10 countries spanning 2009–2022, incidence rates of cancer were associated with worse CKD severity. Incidence rate ratio (IRR) comparing people with an eGFR <60 mL/min/1.73 m^2^ vs ≥60 mL/min/1.73 m^2^ was 1.35 [95% confidence interval (CI) 1.12–1.63, *P *= .002, I^2 ^= 99.9%]. People with eGFR <60 mL/min/1.73 m^2^ were at an elevated rate of cancer compared with eGFR ≥90 mL/min/1.73 m^2^ [IRR 1.48 (95% CI 1.04–2.10, *P *= .03, I^2 ^= 100%)] and those with eGFR 60–89 mL/min/1.73 m^2^ [IRR 1.21 (95% CI 1.11–1.33, *P *< .01, I^2 ^= 92%)]. Age was associated with increased cancer incidence (*β *=* *0.31, *P *= .02) on multiple meta-regression analysis. There was no association between site-specific cancer incidence in CKD patients, but these had wide confidence intervals.

**Conclusion:**

Individuals with CKD have an elevated incidence of cancer, with increasing age contributing to this association. These findings emphasize the importance of investigating whether CKD independently elevates cancer risk, building evidence for tailored cancer screening into CKD patient care.

## Graphical Abstract

**Graphical Abstract:**
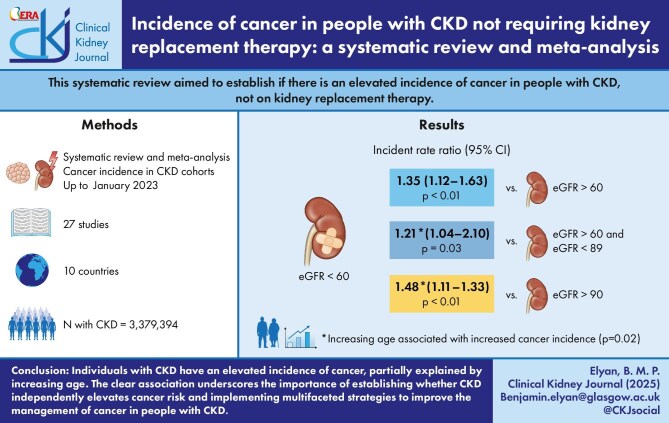

KEY LEARNING POINTS **What was known:**

• Chronic kidney disease (CKD) and cancer share common risk factors and have a bidirectional relationship.
• CKD independently increases cancer mortality.
• Cancer incidence in non-dialysis CKD is inconclusive, with varying evidence on the risk of cancers from multiple patient cohorts.

**This study adds:**

• This meta-analysis shows a 35% higher cancer incidence in non-dialysis CKD patients (estimated glomerular filtration rate <60 mL/min/1.73 m^2^).
• No significant increase in site-specific cancer incidence.
• The elevated cancer risk is not fully explained by age or comorbidities.

**Potential impact:**

• The findings highlight the need of investigating whether CKD independently elevates cancer risk, tailored cancer screening strategies in CKD patients, addressing challenges like imaging limitations and comorbidity burdens.
• Improved risk stratification and inclusion of CKD patients in cancer trials are crucial to optimizing cancer management and reducing mortality in this high-risk population.

## INTRODUCTION

Chronic kidney disease (CKD) is established as an escalating global health challenge, estimated to affect 10% of individuals worldwide [1]. In parallel, cancer stands as a significant contributor to the global healthcare burden, exerting a substantial impact on morbidity and mortality [2]. Understanding and addressing cancer in people with CKD is crucial for improving their overall health outcomes [3].

Concomitant CKD and cancer are common [4], with an established bidirectional relationship for their development [5]. Factors for this relationship include overlapping risk factors (e.g. smoking, obesity), specific cancers that can cause CKD, such as myeloma, risk of CKD from cancer treatments and increased cancer risk from immunosuppressive therapies used in CKD. Comorbidities that commonly accompany CKD, including diabetes and cardiovascular disease, are associated with an in increased risk of cancer [3, 5]. The prevalence of CKD is higher in people with cancer [6] which has implications for the method and likelihood of cancer investigation, treatment choices [7] and recruitment to trials [7]. Importantly, CKD is an independent risk factor for increase in hazards of death from cancer [8].

The link between increased cancer incidence and people with end-stage kidney disease requiring dialysis or kidney transplantation is well established [9–11]. Despite the rising global health burden of CKD, the incidence risk of cancer in people with less severe CKD who do not require kidney replacement therapy (KRT) remains inadequately characterized. Studies reporting cancer event rates in people with CKD (not requiring KRT) are inconclusive as to the degree of the risk of cancer compared with the general population [11]. Some studies have suggested that people with CKD could be at an elevated risk of site-specific cancers, such as urinary tract cancers [11–13]. The presence of albuminuria appears to be independently associated with an increased risk of overall cancer incidence [14] and some site-specific cancers [12, 14, 15]. Furthermore, the landscape and scale of cancer incidence risk in people with CKD not requiring KRT is continually changing, in part because of improved treatment of CKD and conditions associated with CKD, public health policies and shifting population demographics [16]. Delineating whether people with CKD are at elevated risk of cancer incidence is vital for the direction of public health resources, cancer risk prediction and screening, particularly as people with CKD are at an elevated mortality from cancer death [8].

The aims of this systematic review were to identify the available studies that report cancer incidence in people with CKD and establish whether there is an elevated incidence of cancer.

## MATERIALS AND METHODS

This systematic review was conducted in accordance with the Preferred Reporting Items for Systematic Revies and Meta-Analysis (PRISMA) guidelines [17] and was registered on PROSPERO (CRD 42022359690) [18].

### Selection criteria for studies

Studies conducted in adult populations (>18 years of age) that reported cancer incidence in people with CKD were included. Articles were included if cancer incidence was not reported but could be calculated or hazards of developing cancer were reported. Any design of observational or clinical trial was included. Studies examining only patients with end-stage kidney disease on any form of dialysis or renal transplant were excluded from this review. Studies of specialist restricted populations for example with significant comorbidity other than CKD were also excluded.

### Data sources and search strategy

We identified people with CKD stages 3–5 and/or with an estimated glomerular filtration rate (eGFR) <60 mL/min/1.73 m^2^ worldwide that were followed up and subsequently reported cancer incidence. CKD was defined as an eGFR <60 mL/min/1.73 m^2^ for >3 months and characterized by one or more abnormalities of either the function or the structure of the kidney resulting in health implications as per the Kidney Disease: Improving Global Outcomes (KDIGO) criteria [19]. Estimation of GFR using either Modification of Diet in Renal Disease (MDRD) study equation or Chronic Kidney Disease Epidemiology Collaboration (CKD-EPI) equation was accepted. Diagnosis of CKD by relevant diagnostic codes, for example International Statistical Classification of Diseases and Related Health Problems 10th Revision (ICD-10), was also accepted. Cancer diagnoses by relevant diagnostic codes, ICD-10 codes C00–C97 were included. We reported overall cancer incidence excluding non-melanomatous skin cancer (C44).

### Data extraction

Electronic searches (up to and including 17 January 2023) were conducted in PubMed, Embase and the Cochrane Library databases. The search strategy consisted of free text words and Medical Subject Headings (MeSH) terms (Supplementary data, Methods S1). References of associated systematic reviews and included studies were searched, along with grey literature. Contact was made with authors for additional details or clarification where required.

Data were extracted independently by two reviewers (B.M.P.E. and B.T.). using the Rayyan Software [20]. Any disagreements were resolved by a third reviewer (S.B.). Outcome measurements were directly uploaded from imported study tables and standardized using via the TableTidier software [21]. The extraction was guided by the Population, Intervention, Comparison, Outcomes and Study (PICOS) framework allowing reproducibility [22]. A comprehensive list of the data extracted is available in the Supplementary data, Methods S2.

### Quality and risk of bias assessment in individual studies

Two reviewers (B.M.P.E. and B.T.) independently assessed study quality using the Newcastle-Ottawa tool (Supplementary data, Methods S3). This tool has been developed to assess quality of non-randomized studies. According to this ‘star system’, the quality of a study is graded based on its risk of bias in three areas: selection, comparability between groups and outcome assessment. The highest score available is 9 stars signifying a high-quality and low risk of bias study, though any score of 7 stars and above is of high quality [23].

### Data synthesis and analysis

Multiple meta-analyses were performed dictated by the eGFR categories and cancer incidence rates (IRs) reported in the studies. Where stratification of eGFR categories was too heterogeneous for meta-analysis, IRs were pooled, weighted by total follow-up of each category. This was carried out for studies that reported the incidence for all cancer types (excluding non-melanomatous skin cancer) and then subgroup analysis for specific cancer sites, lung, kidney, melanoma, breast, prostate, cervical and urothelial. Variability of effect estimates (IRs) due to between-study heterogeneity was estimated using Higgin and Thompson I^2^, with >75% considered to have a high level of variation in reported incidence due to between-study heterogeneity [24]. Multivariable meta-regressions for patient baseline characteristics including age, sex, year of publication and geographic location were performed.

### Statistical analysis

Analyses were completed using R software version 2023.06.1 [25]. Generalized linear mixed models employing random effects using were chosen for analysis, as the assumption was that cancer incidence would differ between the populations included in the studies due to uncaptured variation. Meta-analyses of cancer IRs and incidence rate ratios (IRRs) were conducted using the inverse variance method with the ‘metarate’ and ‘metainc’ function from the Meta [26] package. The IRR was calculated for each study individually then reporting pooled estimates. Outputs from this package were displayed in forest (forest.meta function), bubble (bubble.metareg) and funnel (funnel function) plots. Model fit was evaluated through log-likelihood, deviance and Akaike Information Criterion.

## RESULTS

### Characteristics of included studies

**Figure 1:**
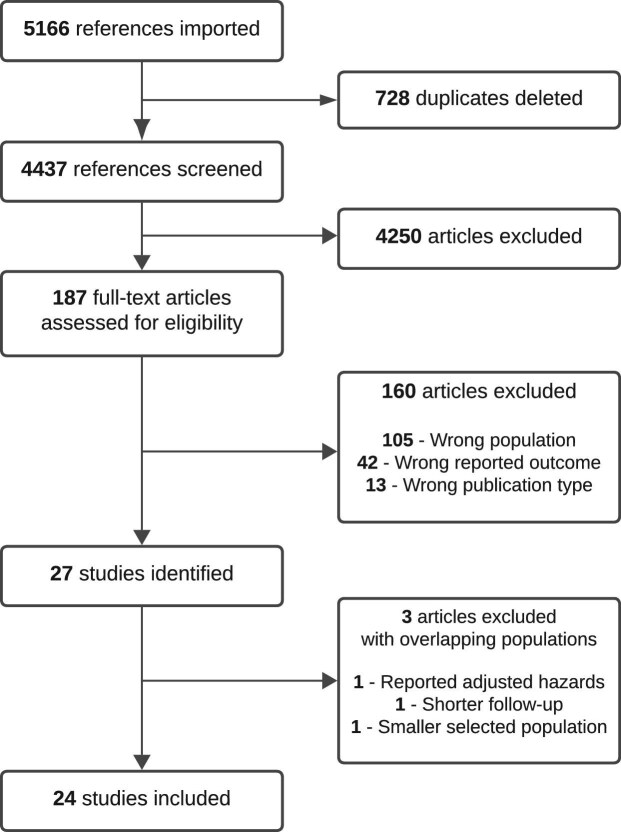
PRISMA diagram.

The search of CENTRAL, MEDLINE and Embase identified 4439 articles (Fig. 1). Of these studies, 27 studies met the inclusion criteria, including 5 519 778 people with CKD, for a total follow-up time of 56 016 681 years from 10 countries. Five studies reporting incidence of all, or the same cancer site had overlapping follow-up periods from the same geographical area [15, 27–30]. We excluded three of these five studies, one that reported adjusted hazards of cancer only [29], another that had shorter follow-up [15] and one with a smaller prospective population selected [28]. IRs of any cancer site with available follow-up times were reported in 20/24 studies (Table 1).

**Table 1:** Overview of studies identified for meta-analysis.

Authors	Year	Randomized Y/N	Study design	Country	Start date	End date	Sample size (*n*)	Cancer site(s)	CKD definition	eGFR categories	Proteinuria analysis
Cancer IRs reported											
Chang *et al*.	2018	N	Retrospective	Taiwan	01/01/1996	31/12/2015	881 430	Gynae and breast cancer	ICD-9-CM 58 and 40	>/<60	x
Chen *et al*. [40]	2016	N	Retrospective	Taiwan	01/01/1997	31/12/2011	1 000 000	Upper tract urothelial carcinoma	MDRD equation	>/<60	x
Choi *et al*.	2022	N	Retrospective	South Korea	2009	31/12/2017	10 505 818	Multiple myeloma	MDRD equation	>/<60, >120, 119–90, 89–60, 59–30, <30	Dipstick
Christensson *et al*.	2013	N	Prospective	Sweden	1974	2006	33 346	All	CKD-EPI creatinine equation 2009	>/<60	x
Chuang *et al*. [41]	2021	N	Retrospective	Taiwan	2000	31/12/2015	471 669	Urothelial carcinoma	CKD-EPI creatinine equation 2009	>90, 89–60, 59–45, <45	Dipstick
Engel *et al*. [31]	2017	Y	Prospective	International	01/12/2008	01/07/2012	14 671	All cancer (other than non-melanoma skin cancers)	Not reported	>/<60	x
Kitchlu *et al*. [34]	2022	N	Retrospective	Canada	01/04/2007	31/12/2017	6 246 941	All	CKD-EPI creatinine equation 2009	>60, 59–45, 44–30, 30–15, <15	x
Lees *et al*. [12]	2021	N	Prospective	UK	2007/2010	2020/2017	502 493	All cancer (other than non-melanoma skin cancers)	CKD-EPI creatinine equation 2009	>90, 89–60, <60	uACR
Liu *et al*.	2020	N	Prospective	China	2011	2015	17 708	All cancer except minor skin cancer	CKD-EPI creatinine equation 2009	>90, 89–60, <60	x
Lowrance *et al*. [30]	2014	N	Retrospective	USA	01/01/2000	01/12/2008	1 190 538	Renal and all except minor skin cancer	CKD-EPI creatinine equation 2009	>120, 119–90, 89–60, 59–30, <30	x
Miyamoto *et al*.	2022	N	Prospective	Japan	1998	31/12/2013	24 593	All except minor skin cancer	modified IDMS–MDRD Study equation and the new Japanese equation	>90, 89–60, 59–45, <45	Dipstick
Oh *et al*.	2020	N	Retrospective	South Korea	2002	31/12/2013	514 795	Colorectal cancer	ICD codes: ‘N18’,‘N19’ ,‘I12’, ‘I13’ ,‘E10.2’, ‘E11.2’, ‘E13.2’ and ‘E14.2’	>/<60	x
Park *et al*. [35]	2021	N	Retrospective	South Korea	2009	31/12/2016	10 505 818	Kidney cancer	MDRD equation	>120, 119–90, 89–60, 59–30, <30	Dipstick
Park *et al*. [27]	2019	N	Retrospective	South Korea	2009	2016	18 936 885	All cancer	MDRD equation	>/<60, >120, 119–90, 89–60, 59–30, <30	x
Sung *et al*. [2]	2022	N	Retrospective	Taiwan	1999	2016	4 578 976	HCC	Not reported	>/<60	x
Wang *et al*.	2017	N	Retrospective	Taiwan	01/01/2000	2013		NMSC	CKD-EPI creatinine equation 2009	>/<60, <15	x
Wong *et al*. [9]	2009	N	Retrospective	Australia	1993	2004	3448	All except minor skin cancer	MDRD equation	>/<60	x
Wong *et al*. [36]	2012	Y	Prospective	International	01/06/2001	01/06/2006	11 140	Colorectal cancer, lung, prostate, urinary tract, breast and skin cancers	MDRD equation	>/<60	x
Wu *et al*.	2013	N	Retrospective	Taiwan	01/01/2004	31/12/2006	96 843	Colorectal cancer	ICD-9-CM codes	>/<60	x
Xu *et al*. [13]	2019	N	Prospective	Sweden	01/01/2006	31/12/2012	1 375 156	All cancer	CKD-EPI creatinine equation 2009	>120, 119–90, 89–60, 59–30, <30	x
Do not report stratified IRs											
Er *et al*.	2016	N	Retrospective	Taiwan	01/01/2005	31/12/2013	985 219	Pancreatic cancer	renal insufficiency	>/<60	x
Oh *et al*.	2018	N	Retrospective	South Korea	2002	2013	1 025 340	Digestive cancer	ICD codes: ‘N18’,‘N19’ ,‘I12’, ‘I13’ ,‘E10.2’, ‘E11.2’, ‘E13.2’ and ‘E14.2’	>/<60	x
Tu *et al*.	2018	N	Prospective	Taiwan	1996	31/12/2008	405 878	All cancer	National Kidney Foundation criteria	>90, 89–60, <60	x
Yu *et al*.	2014	N	Prospective	Taiwan	01/07/1996	01/06/2003		Hepatobiliary cancer, colorectal cancer and lung cancer	CKD-EPI creatinine equation 2009	>/<60	x
Not included due to overlapping follow-up periods and geographical location											
Hoang *et al*. [28]	2020	N	Prospective	South Korea	01/10/2007	31/12/2016	13 644	All	MDRD equation	>90, 89–60, <60	x
Mok *et al*. [15]	2017	N	Prospective	South Korea	01/01/1996	31/12/2012	430 920	Any cancer	CKD-EPI creatinine equation 2009	>90, 89–60, 59–45, <45	Dipstick
Tendulkar *et al*. [29]	2022	N	Retrospective	USA	01/01/2001	1/1/2001	1/12/2020	All cancer (other than non-melanoma skin cancers)	MDRD equation	>60, 59–45, 44–30, <30	x

Y, yes; N, no; uACR, urine albumin-to-creatinine ratio.

There was considerable heterogeneity of eGFR categories included for each study, with 15 reporting the incidence of cancer for people with an eGFR ≥60 mL/min/1.73 m^2^ vs <60 mL/min/1.73 m^2^, and others reporting incidence by more granular eGFR categories.

### IR of all cancers

The IRs of all cancer sites and relevant follow-up times in cohorts with available kidney function assessment were reported in 11/24 studies (9 714 537 people, 53 282 881 person-years).

**Figure 2:**
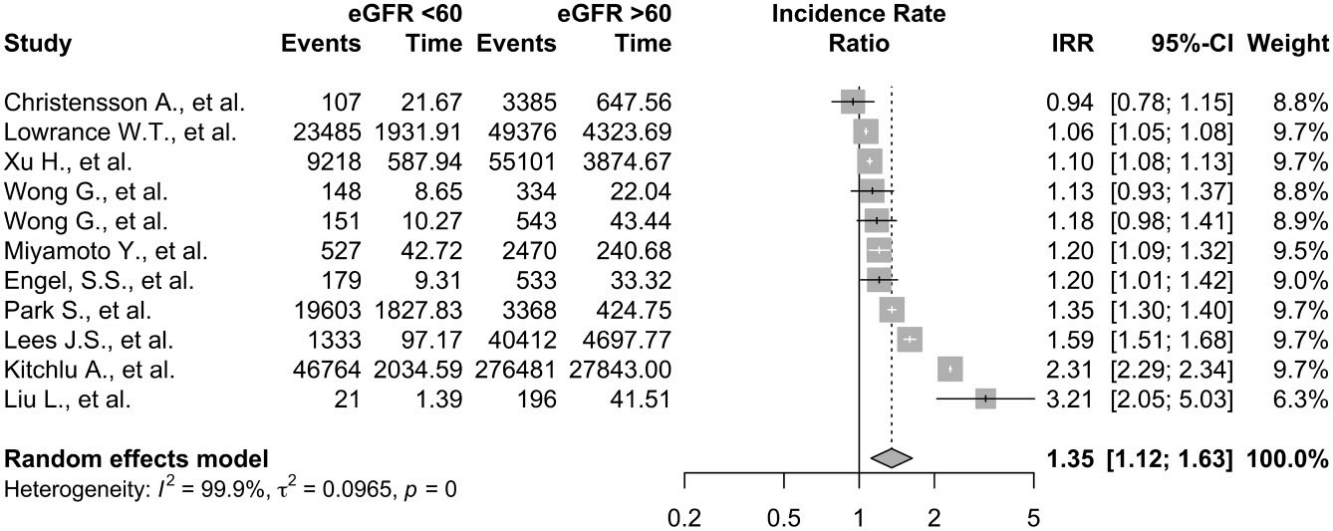
Forest plot showing the pooled IRRs for people with eGFR <60 mL/min/1.73 m^2^ vs ≥60 mL/min/1.73 m^2^.

The overall cancer IRs were 12.38 per 1000 patient-years [95% confidence interval (CI) 10.45–14.32, I^2 ^= 100%], for the 11 studies that allowed for pooled events rates of all cancers and follow-up times. Figure 2 displays the incident rate ratios of cancer for the calculated pooled incidence of all cancer in cohorts with an eGFR <60 mL/min/1.73 m^2^ (total follow-up 6 573 446 person-years) and eGFR ≥60 mL/min/1.73 m^2^ (total follow-up 42 192 422 person-years). The incidence of cancer was higher in people with eGFR of <60 mL/min/1.73 m^2^ vs ≥60 mL/min/1.73 m^2^ with an estimated IRR of 1.35 (95% CI 1.12–1.63, *P *= .002). There was a high level of variation in reported incidence due to between-study heterogeneity [24] (I^2 ^= 99.9%).

**Figure 3:**
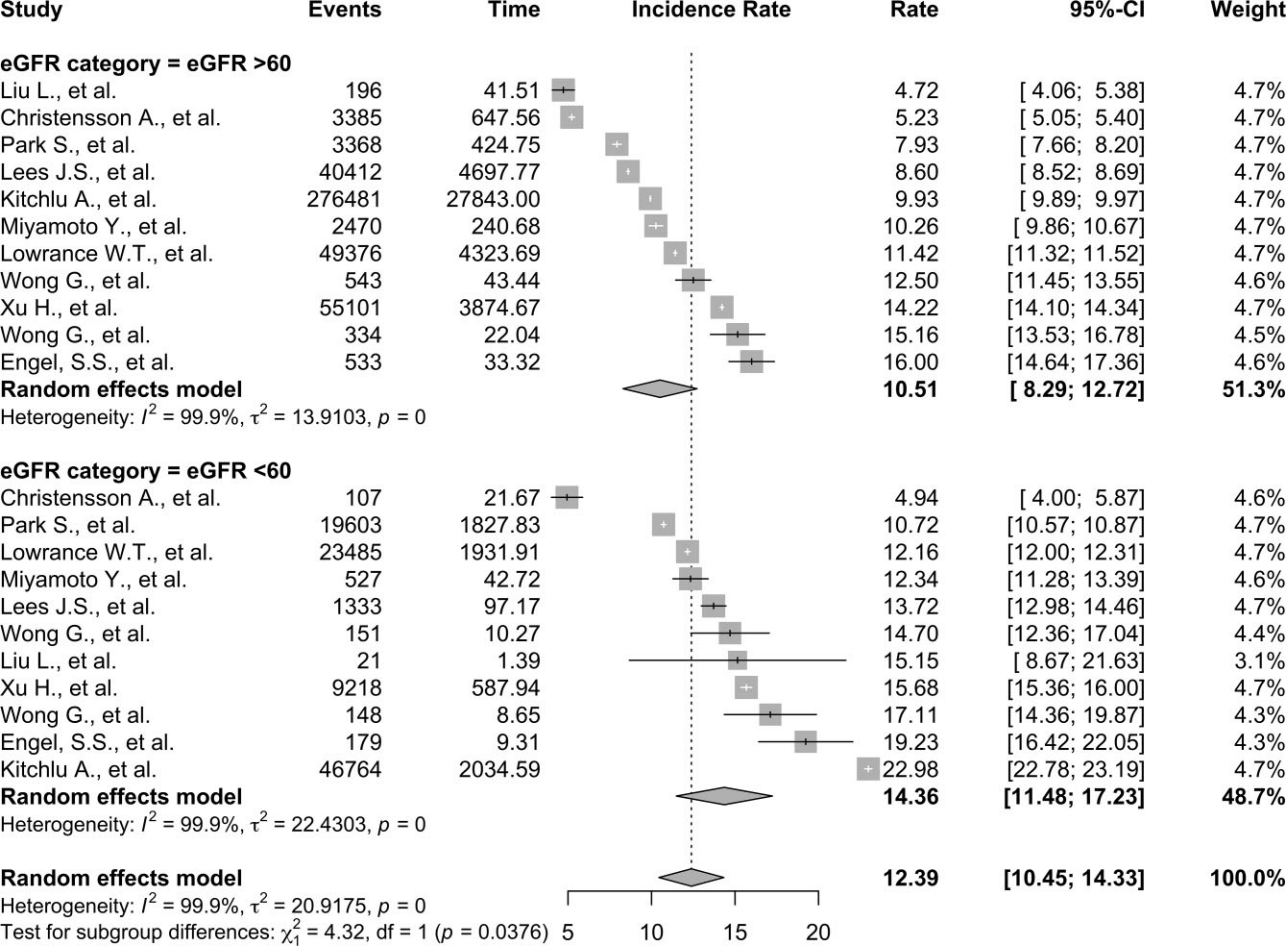
Forest plot showing the pooled IRs per 1000 patient-years for people with eGFR ≥60 mL/min/1.73 m^2^ vs <60 mL/min/1.73 m^2^ to allow for meta-regression analysis.

The IRs of all cancers for people with eGFR ≥60 mL/min/1.73 m^2^ vs eGFR <60 mL/min/1.73 m^2^ were pooled from the studies that reported baseline characteristics for these groups (Fig. 3). The estimated incidence of cancer from these studies was 12.41 per 1000 patient-years (95% CI 10.01–14.81, I^2 ^= 100%). IRs of cancer were reported in people with an eGFR of ≥60 mL/min/1.73 m^2^ in six studies (794 163 people, 5 508 292 person-years) and <60 mL/min/1.73 m^2^ in seven studies (488 482 people, 2 401 036 person-years). The random-effect pooled IRs per 1000 patient-years for the people with eGFR ≥60 mL/min/1.73 m^2^ and <60 mL/min/1.73 m^2^ were similar, measuring 11.40 (95% CI 8.22–14.58 I^2 ^= 100%) and 13.38 (95% CI 9.72–17.05 I^2 ^= 98%), respectively. Multiple meta-regression demonstrated no individual predictor of cancer IR, including sex (*P *= .19), age of the cohort (*P *= .31) or year of publication (*P *= .26) for the examined eGFR groups (Supplementary data, Fig. S1).

**Figure 4:**
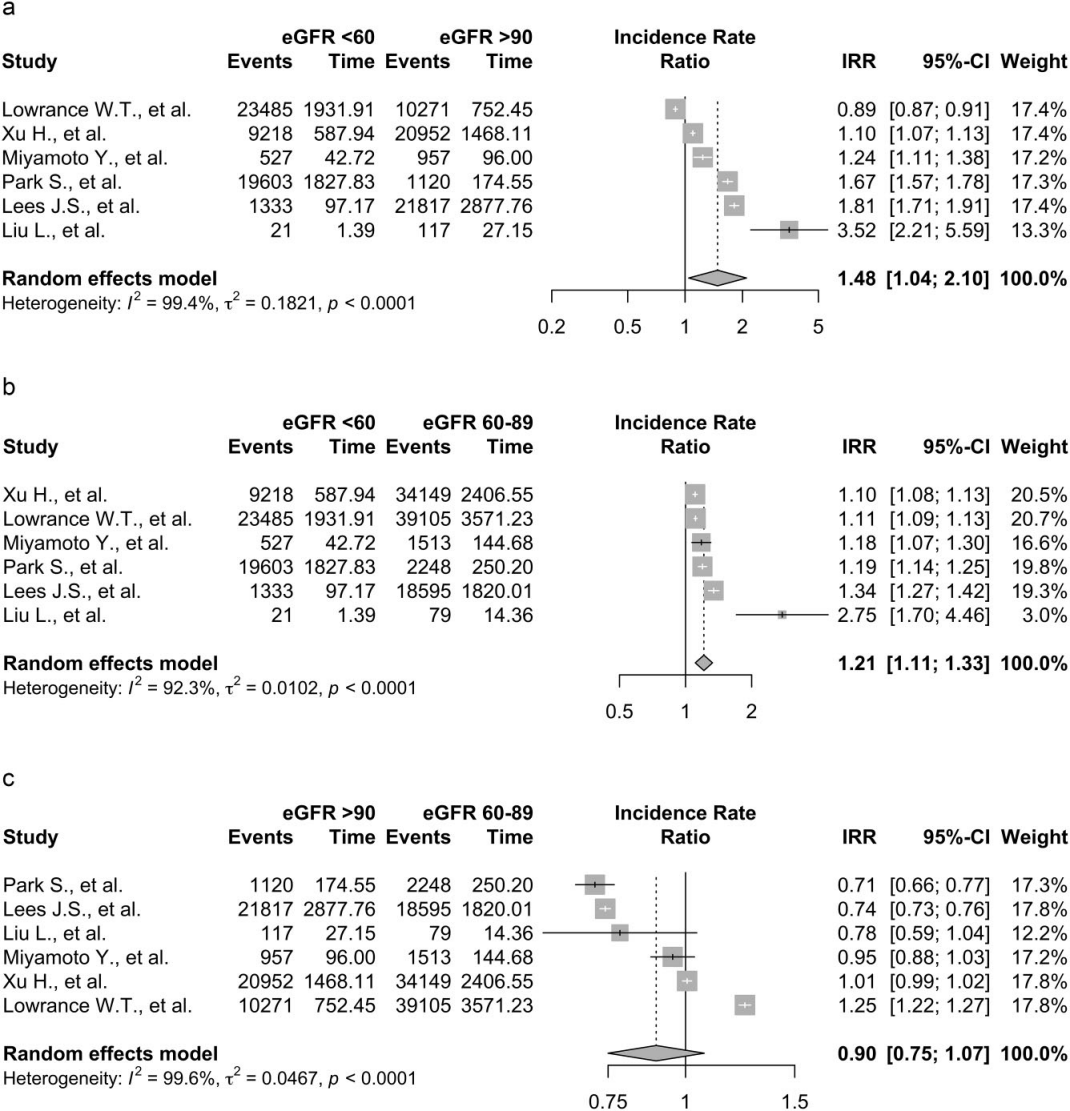
Forest plots showing the pooled IRRs for people with: (**a**) eGFR ≥90 mL/min/1.73 m^2^ vs people with eGFR <60 mL/min/1.73 m^2^. (**b**) eGFR <60 mL/min/1.73 m^2^ vs people with eGFR 60–89 mL/min/1.73 m^2^. (**c**) eGFR ≥90 mL/min/1.73 m^2^ vs people with eGFR 60–89 mL/min/1.73 m^2^.

On pooling cancer IRs in people with an eGFR of ≥90 mL/min/1.73 m^2^ (450 028 people, 4 719 838 person-years), an eGFR of 60–89 mL/min/1.73 m^2^ (643 076 people, 5 796 270 person-years) and <60 mL/min/1.73 m^2^ (488 482 people, 2 401 036 person-years), the cancer IR was 10.77 per 1000 patient-years (95% CI 8.84–12.70, I^2 ^= 100%). Figure 4 displays the pairwise IRRs of cancer for the calculated pooled incidence of all cancer in cohorts with an eGFR of ≥90 mL/min/1.73 m^2^ vs eGFR of 60–89 mL/min/1.73 m^2^ vs eGFR <60 mL/min/1.73 m^2^. The IRR was elevated in people with eGFR <60 mL/min/1.73 m^2^ vs those with eGFR ≥90 mL/min/1.73 m^2^ [IRR 1.48 (95% CI 1.04–2.19, *P *= .03, I^2 ^= 100%)] and vs those with eGFR 60–89 mL/min/1.73 m^2^ [IRR 1.21 (95% CI 1.11–1.33, *P *< .01, I^2 ^= 92%)]. There was no significant difference between the IRR of cancer in those with an eGFR of ≥90 mL/min/1.73 m^2^ vs eGFR of 60–89 mL/min/1.73 m^2^ [IRR 0.90 (95% CI 0.75–1.07, *P *= 0.21, I^2 ^= 100%)].

**Figure 5:**
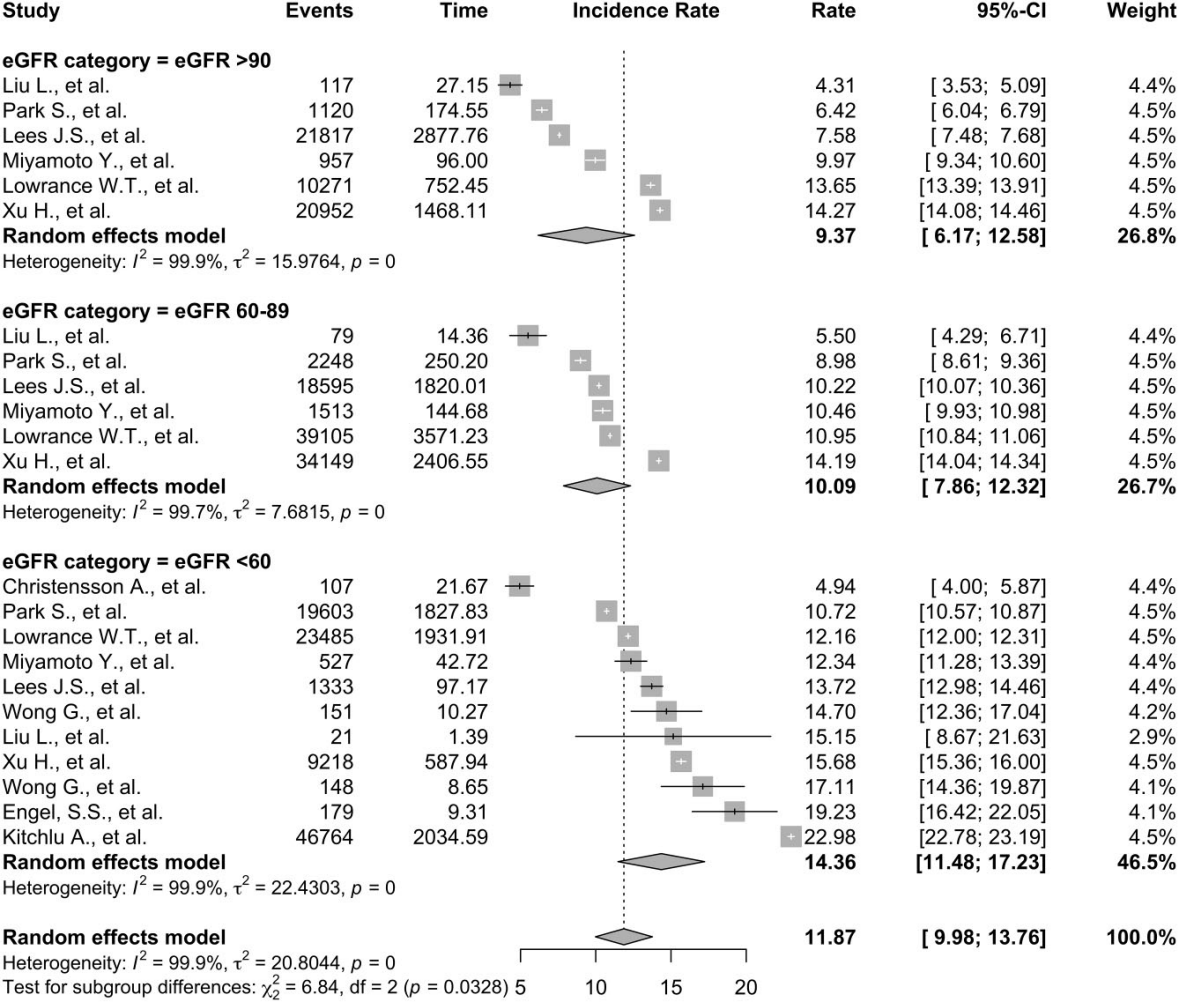
Forest plots showing the pooled IR per 1000 patient-years for people with eGFR <60 mL/min/1.73 m^2^, 60–89 mL/min/1.73 m^2^ and ≥90 mL/min/1.73 m^2^.

To allow for adjustment of baseline characteristics, pooled IRs per 100 patient-years for eGFR subgroups (Fig. 5) for eGFR ≥90 mL/min/1.73 m^2^ was 8.39 (95% CI 5.25–11.53, I^2 ^= 99.8%); for eGFR 60–89 mL/min/1.73 m^2^ was 10.09 (95% CI 7.86–12.32, I^2 ^= 99.7%); and eGFR <60 mL/min/1.73 m^2^ 13.38 (95% CI 9.72–17.04, I^2 ^= 97.9%). Multiple meta-regression accounting for year of publication and age (*P *< .05) exhibited a significant effect on cancer IR (Supplementary data, Fig. S2) but sex did not (*P *= .81).

**Figure 6:**
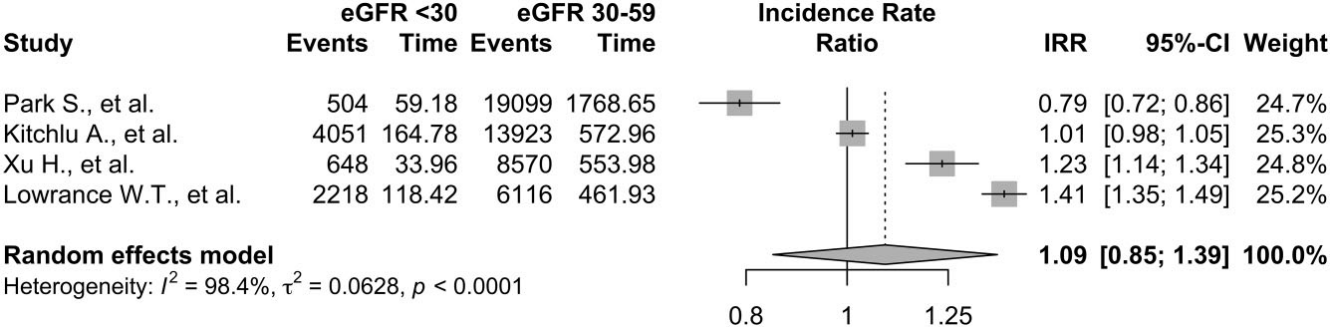
Forest plots showing the pooled IRRs for people with 30–59 mL/min/1.73 m^2^ vs <30 mL/min/1.73 m^2^.

Figure 6 displays the pooled IRRs of all cancers from studies that reported IRs for stratified cohorts of people with an eGFR <60 mL/min/1.73 m^2^. Whilst there was a numerical increase in cancer IR in people with eGFR <30 mL/min/1.73 m^2^ [IR 15.95 (95% CI 10.19–21.71, I^2 ^= 100%)] compared with people with eGFR 30–59 mL/min/1.73 m^2^ [IR 17.72 (95% CI 11.14–24.31, I^2 ^= 100%)], the IRR for people with eGFR < 30 mL/min/1.73 m^2^ was not significantly increased compared with people with eGFR 30–59 mL/min/1.73 m^2^ [IRR 1.09 (95% CI 0.85–1.39, I^2 ^= 98%)].

### IRs of specific cancer sites

The reported IR of cancer stratified by different cancer sites or individual cancer sites was available in 15/24 studies. Meta-analysis of cancer IRs per 1000 person-years in people with CKD (Supplementary data, Figs S3–S10), demonstrated that kidney cancer had an IR of 0.56 (95% CI 0.23–0.89, I^2^* *>* *75%); lung cancer IR 2.09 (95% CI 1.38–2.79, I^2^* *>* *75%); colorectal cancer IR 1.40 (95% CI 1.24–1.56, I^2^* *>* *75%); melanoma IR 2.09 (95% CI 1.38–2.79, I^2^* *>* *75%); breast cancer IR 1.50 (95% CI 0.87–2.13, I^2^* *>* *75%); prostate cancer IR 2.32 (95% CI 1.15–3.49, I^2^* *>* *75%); and urothelial cancer IR 1.05 (95% CI 0.67–1.43, I²* *>* *75%). There was no difference in the cancer IRs across eGFR groups for people with eGFR <60 mL/min/1.73 m^2^ in breast cancer (*P *= .66), urothelial cancer (*P *= .86) and colorectal cancer (*P *= .75), or kidney (*P *= .95), lung (*P *= .83), melanoma (*P *= .83) and prostate cancer (*P *= .98).

### Quality assessment

Study-level assessment for risk of bias across the three domains (selection, comparability, outcome assessment) using the Newcastle-Ottawa score [23] is demonstrated in Supplementary data, Tables S1. This showed that the majority of studies (96%) identified in the search were of high quality and one was moderate [31] quality. The high quality of studies reflects that most of the studies included large, population-based cohorts with baseline characteristics and prolonged follow-up. All people with and without CKD were selected from the same populations but 33% contained cohorts that did not truly or closely represent the general population. Details of the cohort comparability of the cohorts were available in a high proportion (96%) of studies. It should be noted that 33% reported eGFR using the MDRD equation [32] and 37% used the CKD-EPI equation [33], and the remainder did not report the method of eGFR calculation. A high proportion of studies (33%) did not comment on the number or percentage of people lost to follow-up, which is of relevance when reporting cancer incidence.

The study of moderate quality [31] was in the 12 studies that were included in the analysis of all cancer IRs for people with CKD. The IR of all cancers from this study did not differ significantly from the other studies included.

### Publication bias

Neither Begg's test not Egger's test found evidence of publication bias for the meta-analysis of cancer IRRs for the cohorts of people with eGFR ≥60 mL/min/1.73 m^2^ and <60 mL/min/1.73 m^2^, eGFR ≥90 mL/min/1.73 m^2^ and <60 mL/min/1.73 m^2^, or eGFR 60–89 mL/min/1.73 m^2^ and <60 mL/min/1.73 m^2^ (Supplementary data, Fig. S11).

## DISCUSSION

This systematic review pools all the available data on cohorts of individuals with non-dialysis CKD and cancer incidence, demonstrating that people with reduced eGFR (<60 mL/min/1.73 m^2^) experience a 35% higher incidence of cancer to those without (≥60 mL/min/1.73 m^2^) and 58% higher than those with normal kidney function (≥90 mL/min/1.73 m^2^). We found no evidence of increased incidence of site-specific cancer in people with CKD.

Multiple studies have highlighted an elevated cancer incidence in CKD [9, 12, 34, 35] but some have not [36], whilst others have found that this elevated risk is not found in matched population cohorts [27]. This is the first meta-analysis of cancer incidence from observational cohorts in non-dialysis CKD cohorts and builds on previous meta-analyses of patient-level data, also highlighting the elevated risk of cancer in people with CKD [4]. The results from the meta-regression analysis suggest that this is partly explained by an increased age in the cohorts with reduced eGFR. Regardless, these findings highlight that people with reduced eGFR are more commonly diagnosed with cancer and will present a significant challenge with regards to cancer investigation, management and prognosis.

Whether CKD is an independent risk factor for developing cancer is beyond the scope of this review, but we demonstrated that the elevated cancer incidence was not fully explained by adjustment in the multivariable models. It is plausible that people with CKD are highly medicalized and are therefore exposed to excess screening and diagnosis. However, specific genetic, disease, treatment and patient factors found in people with CKD are likely to independently elevate cancer risk in this population [3] Establishing whether people with CKD have an additive excess risk of cancer compared with the general population is crucial when it comes to allocation of public health resources, appropriately adjusted risk prediction and screening programmes. It should be noted, however, that the application of population wide cancer screening programmes to people with CKD may be challenging because of patient factors related to the CKD, for example the increased calcification on imaging [37] and potential risks of radiological iodine contrast agents [38]. Furthermore, the assumption that the early detection and treatment of cancer in people with CKD improves survival is complicated by the fact that people with CKD often carry a higher comorbidity burden than the general population [7] and are routinely excluded from clinical trials of cancer therapies [39].

People with CKD may be at elevated risk of individual cancer types, which may be driving the increased risk in the populations with reduced kidney function [40, 41]. This study was able to meta-analyse available data on the elevated risk of certain types of cancer. The five most common solid organ cancer types recorded in the USA [42]—breast, prostate, lung, colorectal and melanoma—were analysed, along with cervical cancer, urothelial and kidney cancer due to the availability of data for meta-analysis. There are several plausible factors which may substantiate the elevated risk of site-specific cancer in the CKD population, and specifically certain cancer sites, for example urothelial cancer [3, 43]. However, we did not find elevated IRs of site-specific cancers in people with CKD and so this does not seem to account for the increased cancer incidence found in the people with reduced eGFR in this meta-analysis.

If cancer incidence is elevated in people with CKD, as this meta-analysis demonstrates, and the global prevalence of CKD continues to grow [1, 16] then it is imperative that resources are allocated for detection of cancer and to build evidence for management options in this group. A comprehensive review of improving cancer care for people with CKD highlighted a number of practical recommendations that could have marked positive implications in people with CKD who develop cancer [7]. Of relevance, these included recruitment of people with ‘severe renal insufficiency’ to phase 3 clinical trials, use precise estimations of kidney function and develop clinical trial consortiums to include people with CKD. The landscape of systemic anti-cancer therapies is shifting markedly with new immune agents used an increasingly early stages of cancer [44], however people with reduced kidney function are commonly excluded from clinical trials of these therapies [39]. As we move to an era of personalized medicine it is imperative that we have a sound evidence base for the management of cancer in people with reduced kidney function.

This meta-analysis focuses on creatinine-based measures of kidney function which are known to be flawed in the cancer population [45]. Previous studies have shown a wide disparity of access to certain treatment options because of eGFR or creatinine clearance cut-offs [6] and development of side effects from cancer treatment in people with a large disparity between creatinine- and cystatin-based measurements [46]. The use of creatinine-based measures of kidney function as it is influenced by age, sex, race and external factors such as diet [47]. In the cancer population, serum creatinine can be influenced by tubular creatinine secretion from systemic anti-cancer therapies and sarcopenia [48]. Other markers of kidney function (for example cystatin C [49] or clearance of exogenous filtration markers) have been suggested for use in this population, though neither provide perfect measurements of kidney function. We were not able to meta-analyse alternative measures of kidney function due to a lack of reporting but is an important area of research that could improve risk stratification of cancer investigations and management.

People with CKD who develop cancer are at an independently elevated risk of mortality compared with people without CKD [50] and appears to be incremental with advancing stages of CKD [8, 50]. Undoubtedly, there are overlapping factors that partially explain this elevated mortality but also some individual factors that people with CKD commonly experience [3].

Given our findings and the changing landscape of global CKD prevalence, we urgently need to focus on developing strategies that reduce mortality from cancer in people with CKD, regardless of the independence of additional risk of CKD on cancer incidence and mortality. Tailored proactive screening measures balanced against potential unintended harms, appropriately adjusted prediction models with precise kidney function estimations, prompt re-evaluation of resource allocation and inclusion of people with CKD to clinical trials are just some of the changes that could have meaningful positive impacts on people with CKD and cancer.

Overall, the study offers a comprehensive analysis of cancer incidence in individuals with non-dialysis CKD from large international cohorts. Through meta-analysis techniques the study establishes that people with reduced kidney function have an increased incidence of cancer.

This study has limitations that are important to acknowledge. The heterogeneity of eGFR cut-offs, reporting of hazard ratios and granularity of follow-up for each of these groups affected the inclusion of all studies for meta-analysis. Similar factors limited further subgroup analysis and more complex interactions between CKD and other comorbidities and risk factors to the incidence of cancer. In addition, this study did not differentiate between different eGFR equations (MDRD versus CKD-EPI), which have been reported has having differing precision for accuracy of true GFR [5]. It may be that the increased incidence of some cancers in people with reduced kidney function are because of an increased frequency of interactions with healthcare providers therefore introducing significant detection bias. Finally, there may be an independent elevated risk associated with raised urine albumin/protein excretion, which we were not able to fully assess from these cohorts due to a lack of available data.

## CONCLUSION

This systematic review demonstrates an increased incidence of cancer in individuals with CKD compared with those without. Whilst the findings are partially explained by increasing age, the clear association between elevated cancer IR and CKD underscores the importance of determining whether CKD independently elevates cancer risk. Given the findings, future research and resources should establish whether current cancer screening can be applied to the CKD population, build evidence for tailored management options and advocate for focused public health strategies aimed at combating the challenges of cancer in people with CKD.

## Supplementary Material

sfaf084_Supplemental_File

## Data Availability

The data including the extracted tables from studies reporting cancer incidence in CKD cohorts which support the findings of this study are openly available in repository https://github.com at https://github.com/benelyan1/CKD-and-cancer-incidence
