# Supplementary material for: Incidence of cancer in people with CKD not requiring kidney replacement therapy: a systematic review and meta-analysis
Source: Clin Kidney J. 2025 Mar 21;18(5):sfaf084. doi: 10.1093/ckj/sfaf084 (PMC12086533; doi:10.1093/ckj/sfaf084)
Supplement: sfaf084_Supplemental_File [file sfaf084_supplemental_file.docx]

## **Supplementary Material**

### **Supplementary Methods S1**

PRISMA checklist

| **Section and Topic** | **Item #** | **Checklist item** | **Location where item is reported** |
| --- | --- | --- | --- |
| **TITLE** | | |  |
| Title | 1 | Identify the report as a systematic review. | Title: page1 |
| **ABSTRACT** | | |  |
| Abstract | 2 | See the PRISMA 2020 for Abstracts checklist. | Abstract: page 2 |
| **INTRODUCTION** | | |  |
| Rationale | 3 | Describe the rationale for the review in the context of existing knowledge. | Introduction: page 4 |
| Objectives | 4 | Provide an explicit statement of the objective(s) or question(s) the review addresses. | Introduction: page 5 |
| **METHODS** | | |  |
| Eligibility criteria | 5 | Specify the inclusion and exclusion criteria for the review and how studies were grouped for the syntheses. | Methods: Data Sources and Search Strategy: page 5 |
| Information sources | 6 | Specify all databases, registers, websites, organisations, reference lists and other sources searched or consulted to identify studies. Specify the date when each source was last searched or consulted. | Methods: Data Sources and Search Strategy: page 5 |
| Search strategy | 7 | Present the full search strategies for all databases, registers and websites, including any filters and limits used. | Supplementary Methods S1, pages 1-4 |
| Selection process | 8 | Specify the methods used to decide whether a study met the inclusion criteria of the review, including how many reviewers screened each record and each report retrieved, whether they worked independently, and if applicable, details of automation tools used in the process. | Methods: Selection and Eligibility Criteria: page 6 and 7 |
| Data collection process | 9 | Specify the methods used to collect data from reports, including how many reviewers collected data from each report, whether they worked independently, any processes for obtaining or confirming data from study investigators, and if applicable, details of automation tools used in the process. | Methods: Data extraction: page 7 |
| Data items | 10a | List and define all outcomes for which data were sought. Specify whether all results that were compatible with each outcome domain in each study were sought (e.g. for all measures, time points, analyses), and if not, the methods used to decide which results to collect. | Methods: Selection and Eligibility Criteria: page 6 and 7 Supplementary Methods S2, page 5 |
|  | 10b | List and define all other variables for which data were sought (e.g. participant and intervention characteristics, funding sources). Describe any assumptions made about any missing or unclear information. | Methods: Selection and Eligibility Criteria: page 6 and 7 Supplementary Methods S2, page 4 |
| Study risk of bias assessment | 11 | Specify the methods used to assess risk of bias in the included studies, including details of the tool(s) used, how many reviewers assessed each study and whether they worked independently, and if applicable, details of automation tools used in the process. | Methods: Risk of bias assessment in individual studies: page 7. Supplementary Methods S3, pages 4-6 |
| Effect measures | 12 | Specify for each outcome the effect measure(s) (e.g. risk ratio, mean difference) used in the synthesis or presentation of results. | Methods: Data synthesis and analysis: page 8 |
| Synthesis methods | 13a | Describe the processes used to decide which studies were eligible for each synthesis (e.g. tabulating the study intervention characteristics and comparing against the planned groups for each synthesis (item #5)). | Methods: Selection and Eligibility Criteria: page 6 |
|  | 13b | Describe any methods required to prepare the data for presentation or synthesis, such as handling of missing summary statistics, or data conversions. | Methods: Data extraction: page 7 |
|  | 13c | Describe any methods used to tabulate or visually display results of individual studies and syntheses. | Methods: Data extraction: page 7 |
|  | 13d | Describe any methods used to synthesize results and provide a rationale for the choice(s). If meta-analysis was performed, describe the model(s), method(s) to identify the presence and extent of statistical heterogeneity, and software package(s) used. | Methods: Statistical analysis: page 8 |
|  | 13e | Describe any methods used to explore possible causes of heterogeneity among study results (e.g. subgroup analysis, meta-regression). | Methods: Data synthesis and analysis: page 8 |
|  | 13f | Describe any sensitivity analyses conducted to assess robustness of the synthesized results. | Methods: Data synthesis and analysis: page 8 |
| Reporting bias assessment | 14 | Describe any methods used to assess risk of bias due to missing results in a synthesis (arising from reporting biases). | Methods: Risk of bias assessment in individual studies: page 7. Supplementary Methods S3, pages 4-6 |
| Certainty assessment | 15 | Describe any methods used to assess certainty (or confidence) in the body of evidence for an outcome. | NA |
| **RESULTS** | | |  |
| Study selection | 16a | Describe the results of the search and selection process, from the number of records identified in the search to the number of studies included in the review, ideally using a flow diagram. | Results: Characteristics of included studies: pages 8 and 9 |
|  | 16b | Cite studies that might appear to meet the inclusion criteria, but which were excluded, and explain why they were excluded. | Tables: Table 1 |
| Study characteristics | 17 | Cite each included study and present its characteristics. | Tables: Table 1 |
| Risk of bias in studies | 18 | Present assessments of risk of bias for each included study. | Supplementary Tables S1 |
| Results of individual studies | 19 | For all outcomes, present, for each study: (a) summary statistics for each group (where appropriate) and (b) an effect estimate and its precision (e.g. confidence/credible interval), ideally using structured tables or plots. | Results: pages 9-12 |
| Results of syntheses | 20a | For each synthesis, briefly summarise the characteristics and risk of bias among contributing studies. | Results: pages 9-12 |
|  | 20b | Present results of all statistical syntheses conducted. If meta-analysis was done, present for each the summary estimate and its precision (e.g. confidence/credible interval) and measures of statistical heterogeneity. If comparing groups, describe the direction of the effect. | Results: pages 9-12 |
|  | 20c | Present results of all investigations of possible causes of heterogeneity among study results. | Results: pages 9-12 |
|  | 20d | Present results of all sensitivity analyses conducted to assess the robustness of the synthesized results. | Results: pages 9-12 |
| Reporting biases | 21 | Present assessments of risk of bias due to missing results (arising from reporting biases) for each synthesis assessed. | Results: Quality assessment: pages 11 and 12 |
| Certainty of evidence | 22 | Present assessments of certainty (or confidence) in the body of evidence for each outcome assessed. | Results: Quality assessment: pages 11 and 12 |
| **DISCUSSION** | | |  |
| Discussion | 23a | Provide a general interpretation of the results in the context of other evidence. | Discussion: pages 12-15 |
|  | 23b | Discuss any limitations of the evidence included in the review. | Discussion: page 15 |
|  | 23c | Discuss any limitations of the review processes used. | Discussion: page 15 |
|  | 23d | Discuss implications of the results for practice, policy, and future research. | Discussion: pages 12-15, Conclusion: page 15 |
| **OTHER INFORMATION** | | |  |
| Registration and protocol | 24a | Provide registration information for the review, including register name and registration number, or state that the review was not registered. | Methods: page 6 |
|  | 24b | Indicate where the review protocol can be accessed, or state that a protocol was not prepared. | Methods: page 6 |
|  | 24c | Describe and explain any amendments to information provided at registration or in the protocol. | NA |
| Support | 25 | Describe sources of financial or non-financial support for the review, and the role of the funders or sponsors in the review. | Disclosure statement: page 16 |
| Competing interests | 26 | Declare any competing interests of review authors. | Disclosure statement: page 16 |
| Availability of data, code and other materials | 27 | Report which of the following are publicly available and where they can be found: template data collection forms; data extracted from included studies; data used for all analyses; analytic code; any other materials used in the review. | Data sharing statement: page 16 |

### **Supplementary Methods S2**

### Search strategy

Embase

1., exp kidney failure/

2., ((chronic or dysfunction or failure) adj4 (kidney or renal)).tw.

3., (ckd or crd or ckf or crf).tw.

4., exp glomerulus filtration rate/

5., (low gfr or low glomerular filtration rate or low egfr or low estimated glomerular filtration rate).tw.

6., or/1-5

7., exp neoplasm/

8., exp neoplasms by histologic type/

9., exp cancer staging/

10., (cancer* or neoplas* or tumo?r* or malignan* or carcinoma* or adencarcinoma* or oncolog*).tw.

11., or/7-10

12., Clinical study/

13., exp case control study/

14., Family study/

15., Longitudinal study/

16., Retrospective study/

17., Prospective study/

18., Randomized controlled trials/

19., 6 not 7

20., Cohort analysis/

21., (Cohort adj (study or studies)).mp.

22., (Case control adj (study or studies)).tw.

23., (follow up adj (study or studies)).tw.

24., (observational adj (study or studies)).tw.

25., (epidemiologic$ adj (study or studies)).tw.

26., (cross sectional adj (study or studies)).tw.

27., or/12-16,19-26

28., exp cumulative incidence/ or exp cancer incidence/ or exp incidence/

29., 6 and 11 and 27 and 28

MEDLINE

1. exp Renal Insufficiency, Chronic/
2. ((chronic or dysfunction or failure) adj4 (kidney or renal)).tw.
3. (ckd or crd or crf or ckf).tw.
4. (low gfr or low glomerular filtration rate or low egfr).tw.
5. Glomerular Filtration Rate/
6. 1 or 2 or 3 or 4 or 5
7. exp Neoplasms/
8. (cancer or cancers or neoplasm or neoplasms or tumo?r or tumo?rs or malignancy or malignancies or carcinoma or carcinomas or adenocarcinoma or adenocarcinomas or oncology or oncologies).tw.
9. Neoplasm Staging/
10. or/7-9
11. Epidemiologic studies/
12. exp case control studies/
13. exp cohort studies/
14. Case control.tw.
15. (cohort adj (study or studies)).tw.
16. Cohort analy$.tw.
17. (Follow up adj (study or studies)).tw.
18. (observational adj (study or studies)).tw.
19. Longitudinal.tw.
20. Retrospective.tw.
21. or/11-20
22. exp incidence/
23. (incidence or incidences).tw.
24. (incidence adj (study or studies)).tw.
25. or/22-24
26. 6 and 10 and 21 and 25

CENTRAL

#1 MeSH descriptor: [Renal Insufficiency, Chronic] explode all trees

#2 ((chronic or dysfunction or failure) near/3 (kidney or renal)):ti,ab

#3 (ckd or crd or crf or ckf):ti,ab

#4 (low gfr or low glomerular filtration rate or low egfr):ti,ab

#5 MeSH descriptor: [Neoplasms] explode all trees

#6 (cancer or cancers or neoplasm or neoplasms or tumo?r or tumo?rs or malignancy or malignancies or carcinoma or carcinomas or adencarcinoma or adenocarcinoma or oncology or oncologies):ti,ab

#7 MeSH descriptor: [Neoplasm Staging] explode all trees

#8 MeSH descriptor: [Cohort Studies] explode all trees

#9 (case control):ti,ab

#10 (Follow up near (study or studies)):ti,ab

#11 (observational near (study or studies)):ti,ab

#12 (Longitudinal):ti,ab

#13 (Retrospective):ti,ab

#14 MeSH descriptor: [Case-Control Studies] explode all trees

#15 MeSH descriptor: [Epidemiologic Studies] explode all trees

#16 (cohort near (study or studies)):ti,ab

#17 (cohort analysis):ti,ab

#18 MeSH descriptor: [Incidence] explode all trees

#19 (incidence):ti,ab

#20 {OR #1-#4}

#21 {OR #5-#7}

#22 {OR #8-#17}

#23 {OR #18-#19}

#24 {AND #20-#23}

### **Supplementary Methods S2**

PICOS framework for data collection

| Population | inclusion and exclusion criteria, country, start date follow-up, end date follow-up, duration follow-up, sample size, baseline characteristics of each cohort |
| --- | --- |
| Intervention/Exposure | CKD definition, eGFR categories, eGFR calculation method, proteinuria analysis |
| Comparison | eGFR categories, eGFR calculation, proteinuria analysis |
| Outcome | Cancer incidence, cancer site(s), |
| Study | Authors, publication year, study design randomised cohort, recruitment strategy |

### **Supplementary Methods S3**

Newcastle-Ottawa Scale adapted to cohort studies

**Selection (Maximum 4 stars)**

1. Representativeness of the exposed cohort:
   - Truly representative (one star)
   - Somewhat representative (one star)
   - Selected group
   - No description of the derivation of the cohort
2. Selection of the non-exposed cohort:
   - Drawn from the same community as the exposed cohort (one star)
   - Drawn from a different source
   - No description of the derivation of the non-exposed cohort
3. Ascertainment of exposure:
   - Secure record (e.g., surgical record)
   - Structured interview (one star)
   - Written self-report
   - No description
   - Other (one star)
4. Demonstration that outcome of interest was not present at the start of the study:
   - Yes (one star)
   - No

**Comparability (Maximum 2 stars)**

- Comparability of cohorts on the basis of the design or analysis controlled for confounders:
  - The study controls for age, sex, and marital status (one star)
  - Study controls for other factors (list) (one star)
  - Cohorts are not comparable on the basis of the design or analysis controlled for confounders

**Outcome (Maximum 3 stars)**

1. Assessment of outcome:
   - Independent blind assessment
   - Record linkage (one star)
   - Self-report
   - No description
   - Other (one star)
2. Was follow-up long enough for outcomes to occur:
   - Yes (one star)
   - No
3. Adequacy of follow-up of cohorts:
   - Complete follow up- all subjects accounted for (one star)
   - Subjects lost to follow up unlikely to introduce bias- number lost less than or equal to 20% or description of those lost suggested no different from those followed (one star)
   - Follow up rate less than 80% and no description of those lost
   - No statement

### **Supplementary Figure S1**

Bubble plot of meta-regression analysis for cancer incidence of people with eGFR ≥60mL/min/1.73m^2^ and <60mL/min/1.73m^2^ by:

a) Age of cohort


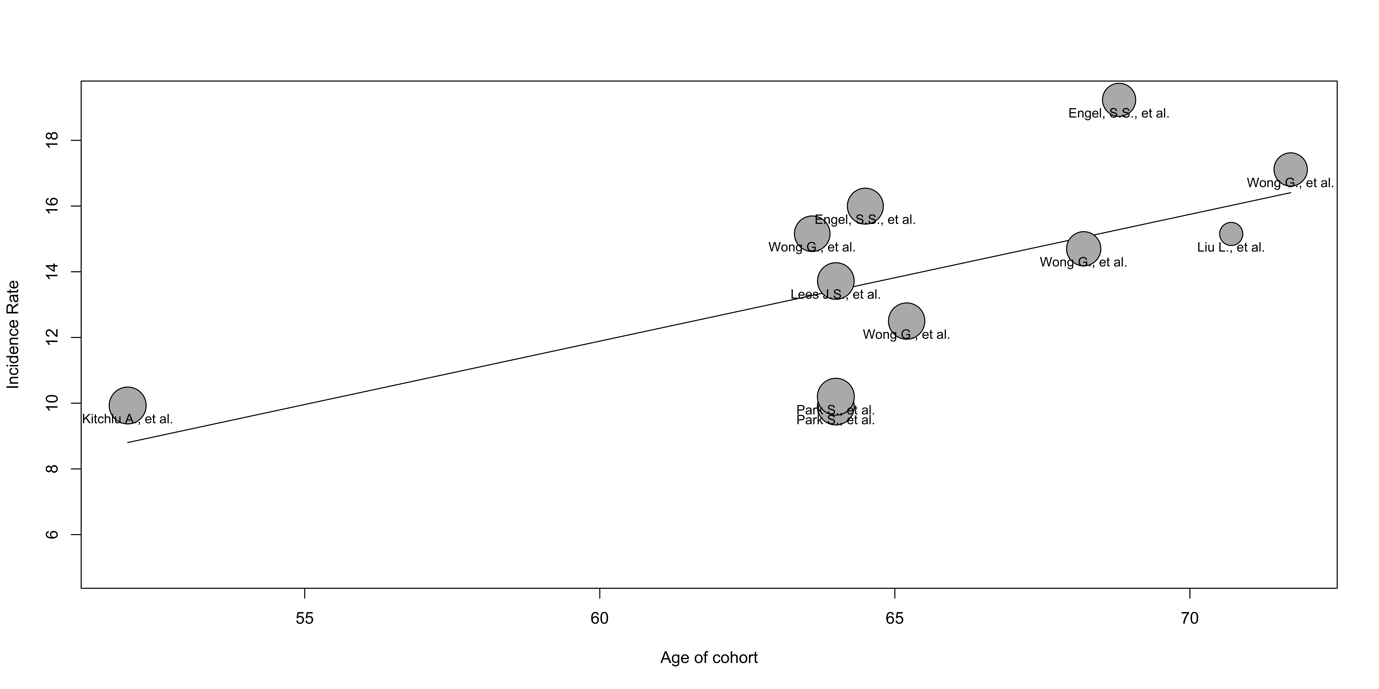


b) Percentage of male sex


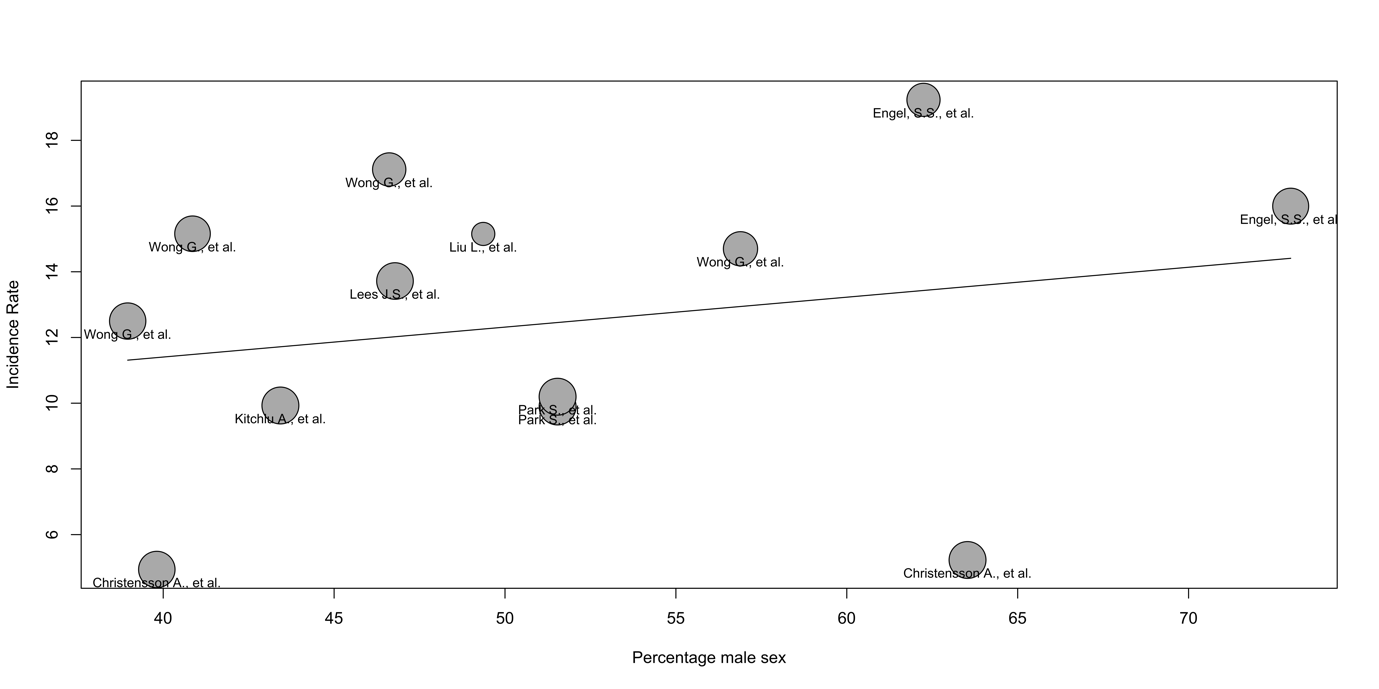


c) Publication year


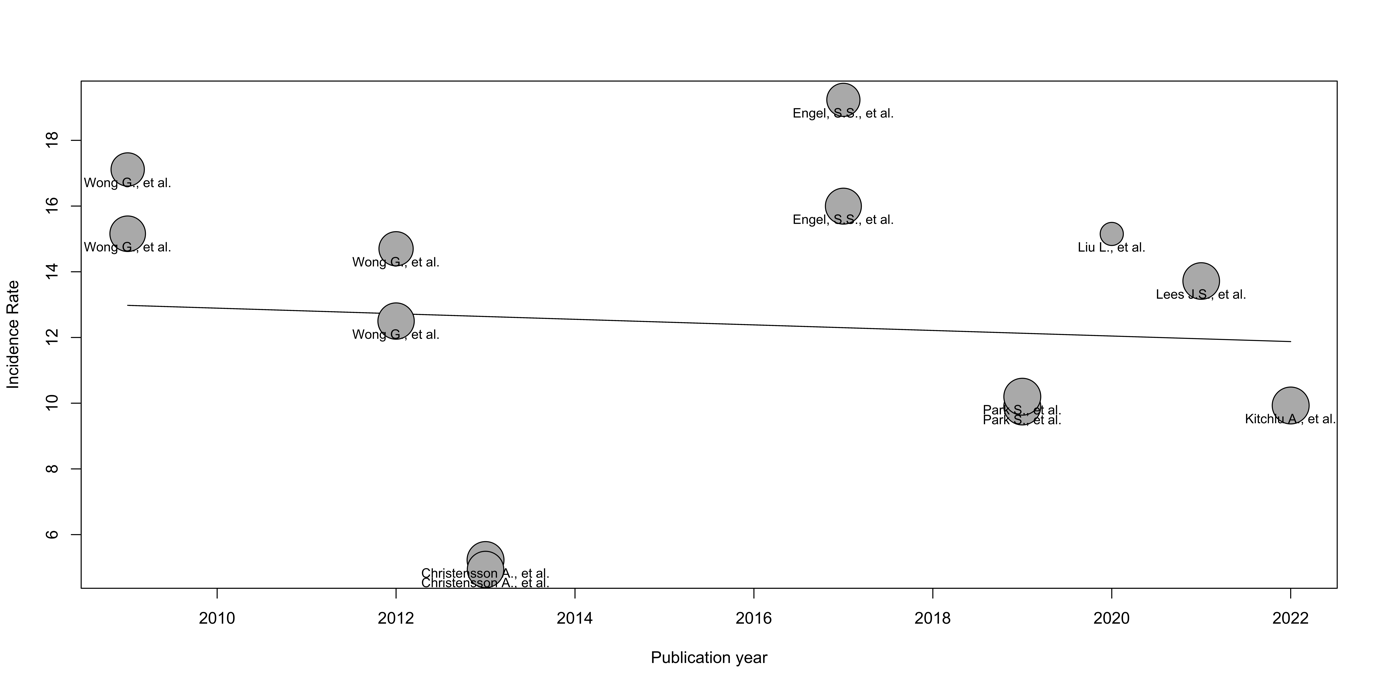


### **Supplementary Figure S2**

Bubble plot of meta-regression analysis for cancer incidence in people with eGFR of ≥90mL/min/1.73m^2^, 60-89mL/min/1.73m^2^ and <60mL/min/1.73m^2^ by:

a) Age of cohort


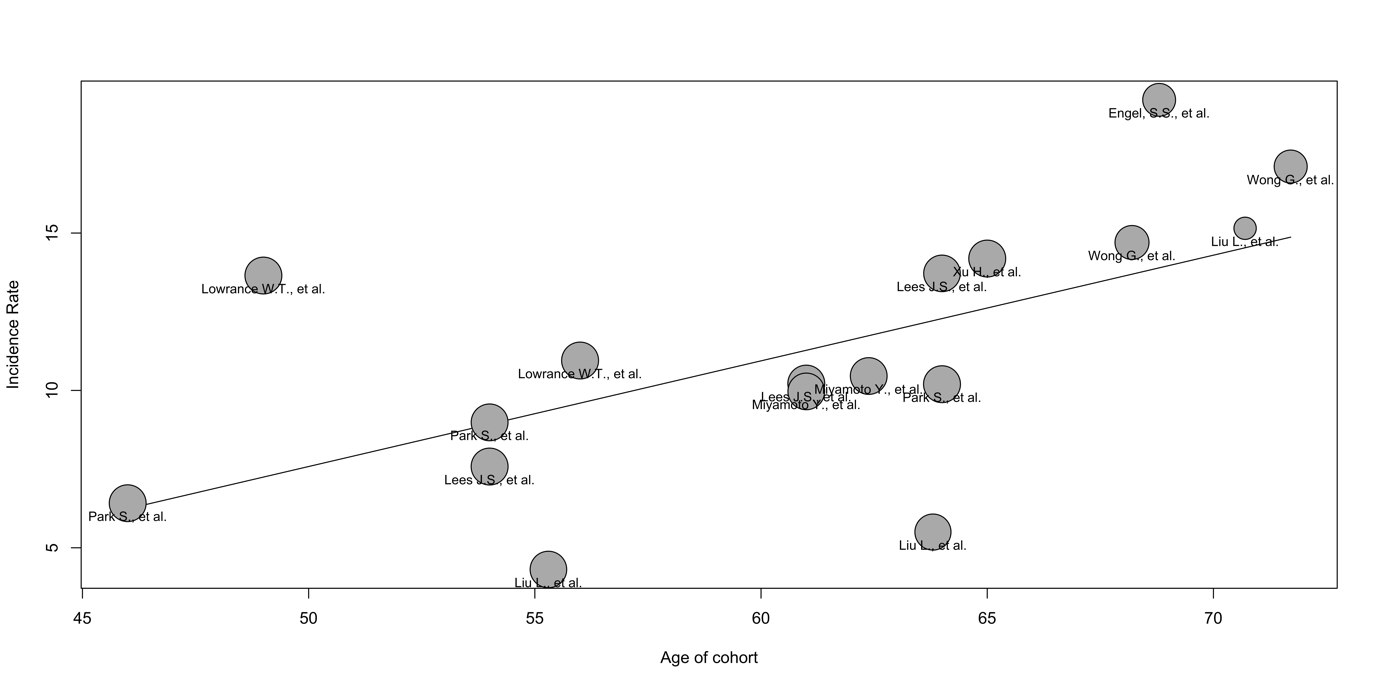


b) Percentage of male sex


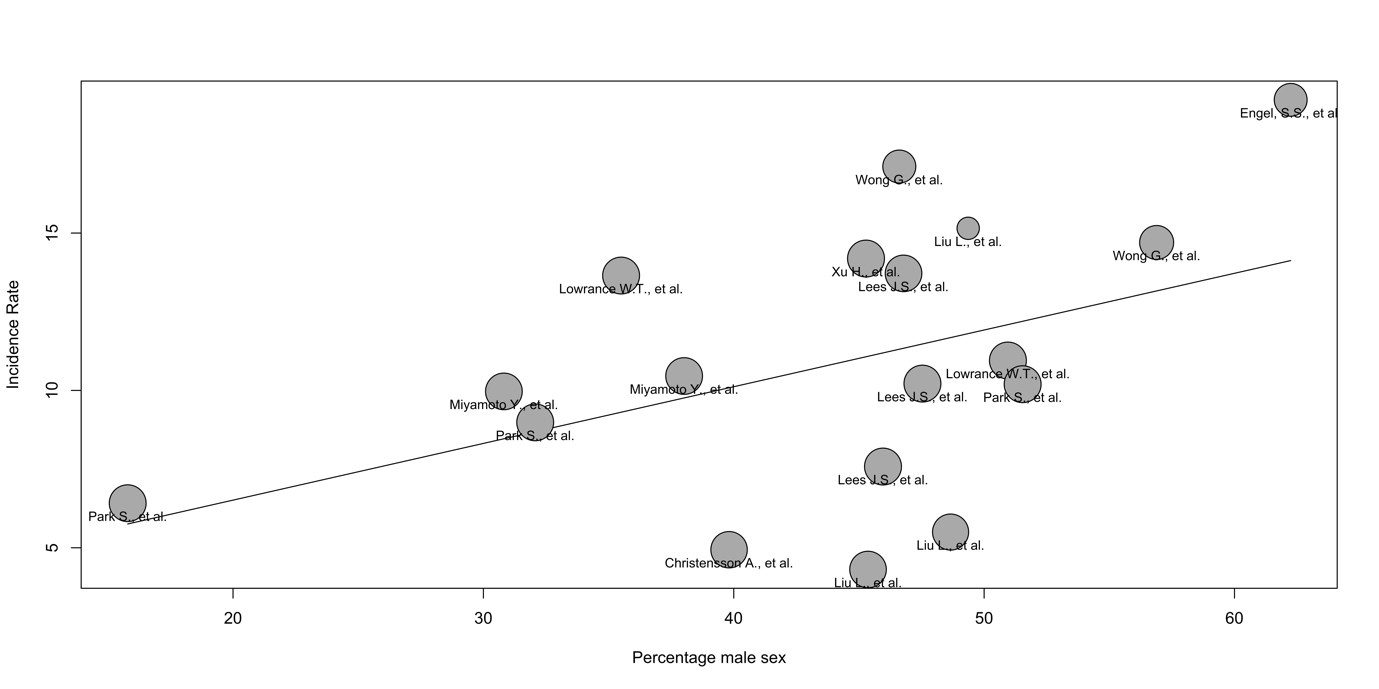


c) Publication year


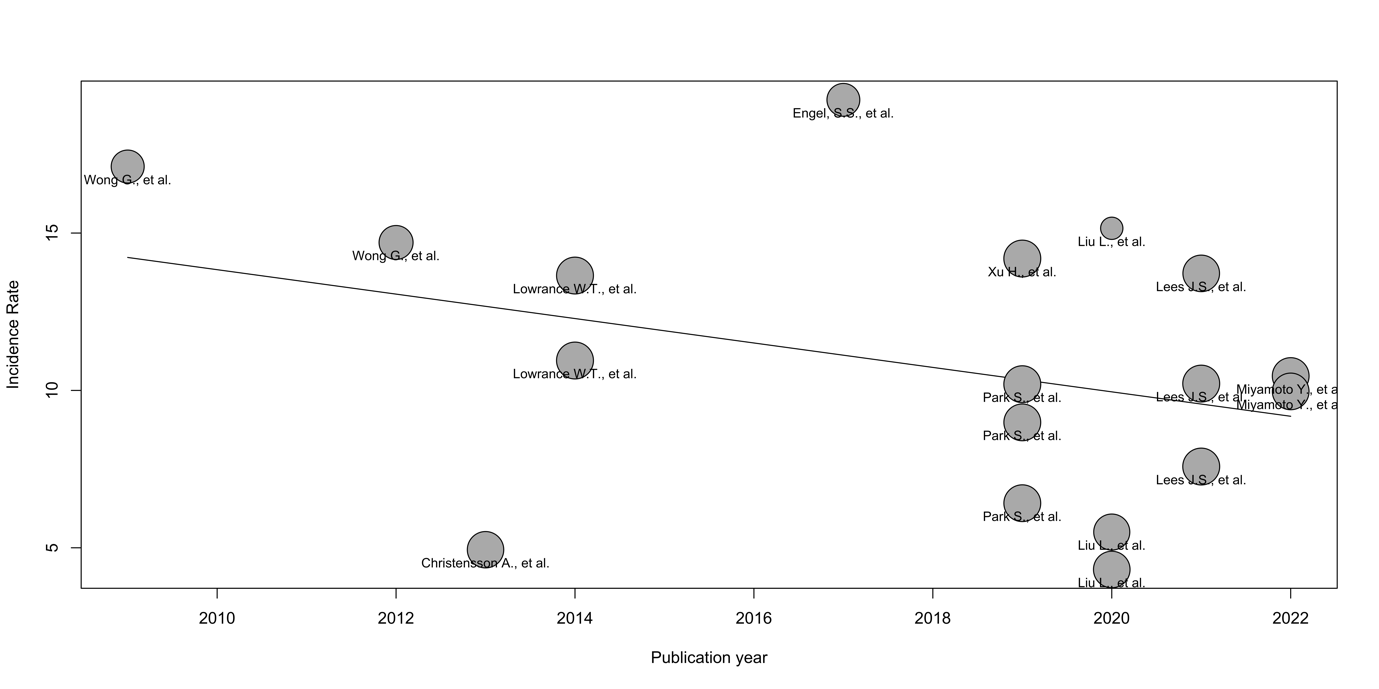


### **Supplementary Figure S3**

Kidney cancer incidence in people with eGFR <60mL/min/1.73m^2^


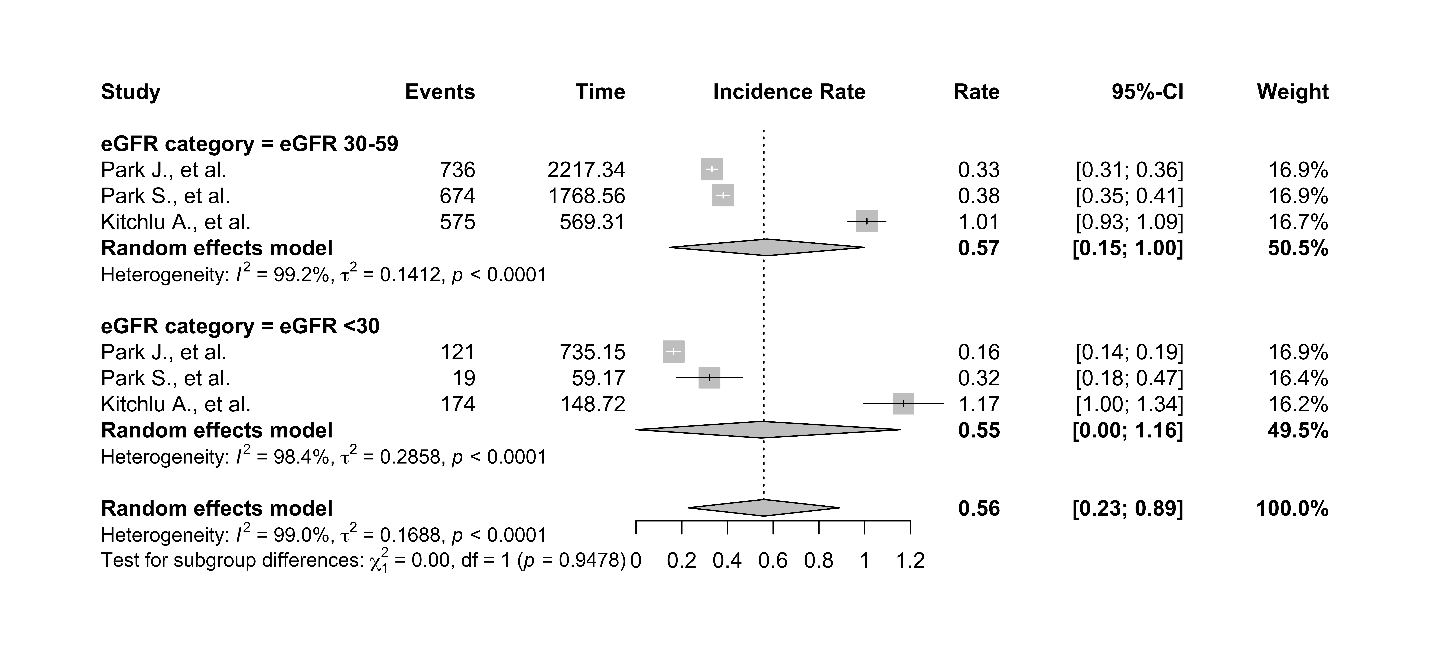


### **Supplementary Figure S4**

Lung cancer incidence in people with eGFR <60mL/min/1.73m^2^


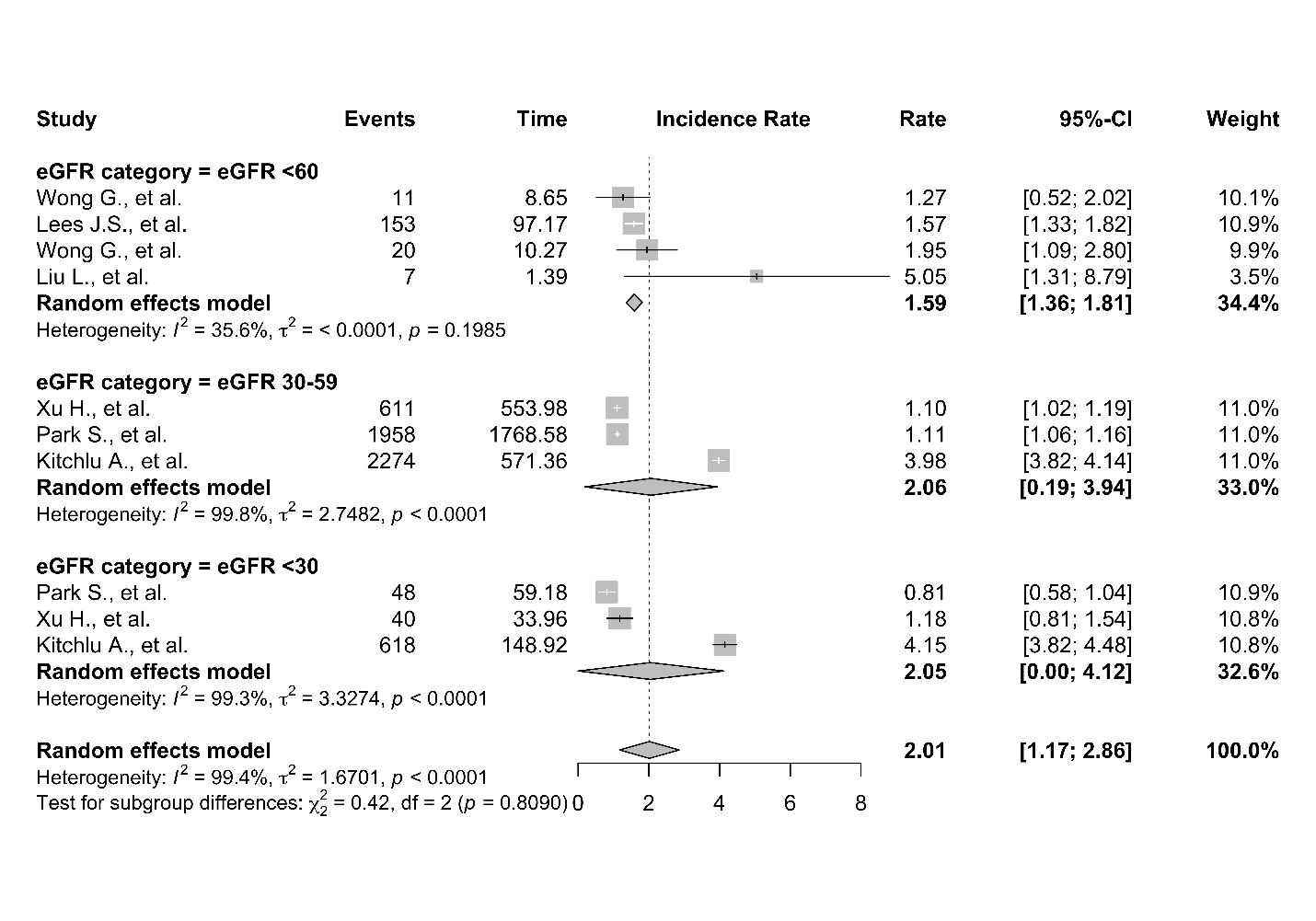


### **Supplementary Figure S5**

Colorectal cancer incidence in people with eGFR <60mL/min/1.73m^2^


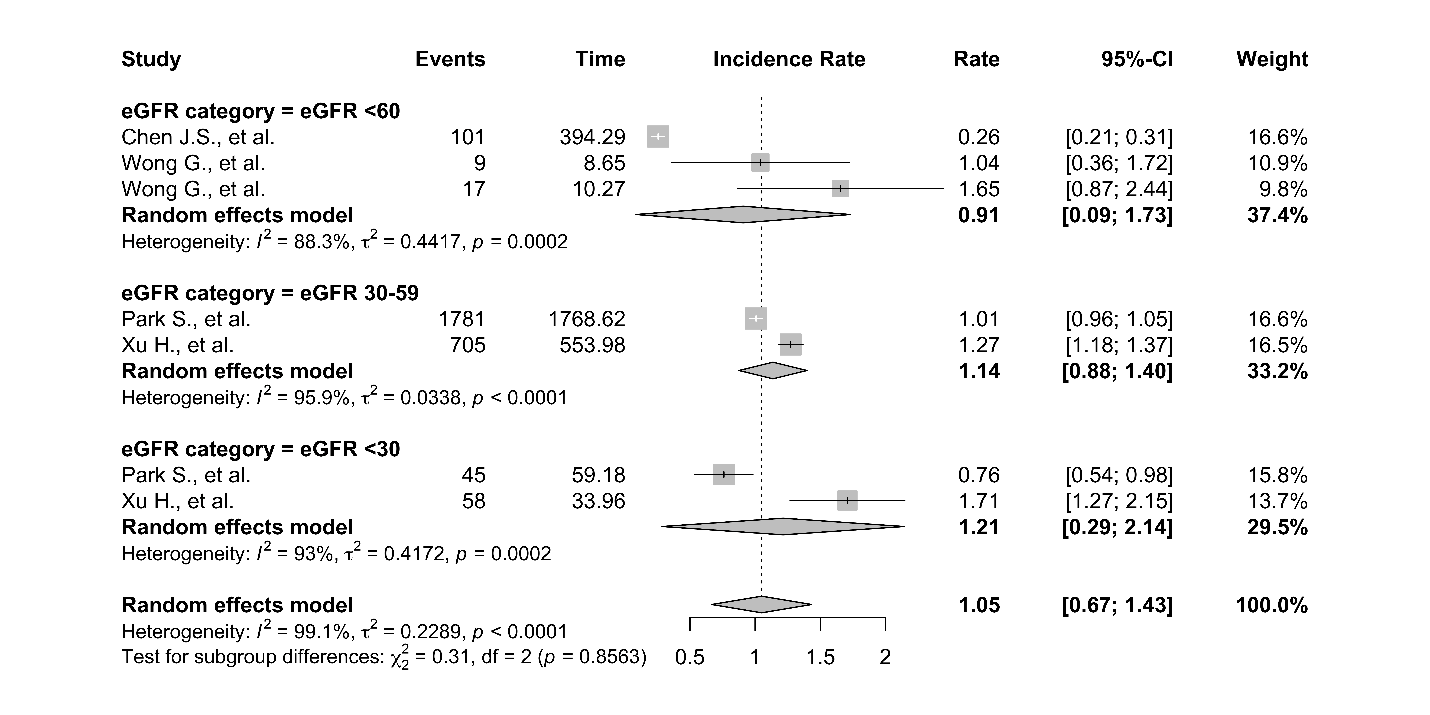


### **Supplementary Figures S6**

Melanoma incidence in people with eGFR <60mL/min/1.73m^2^


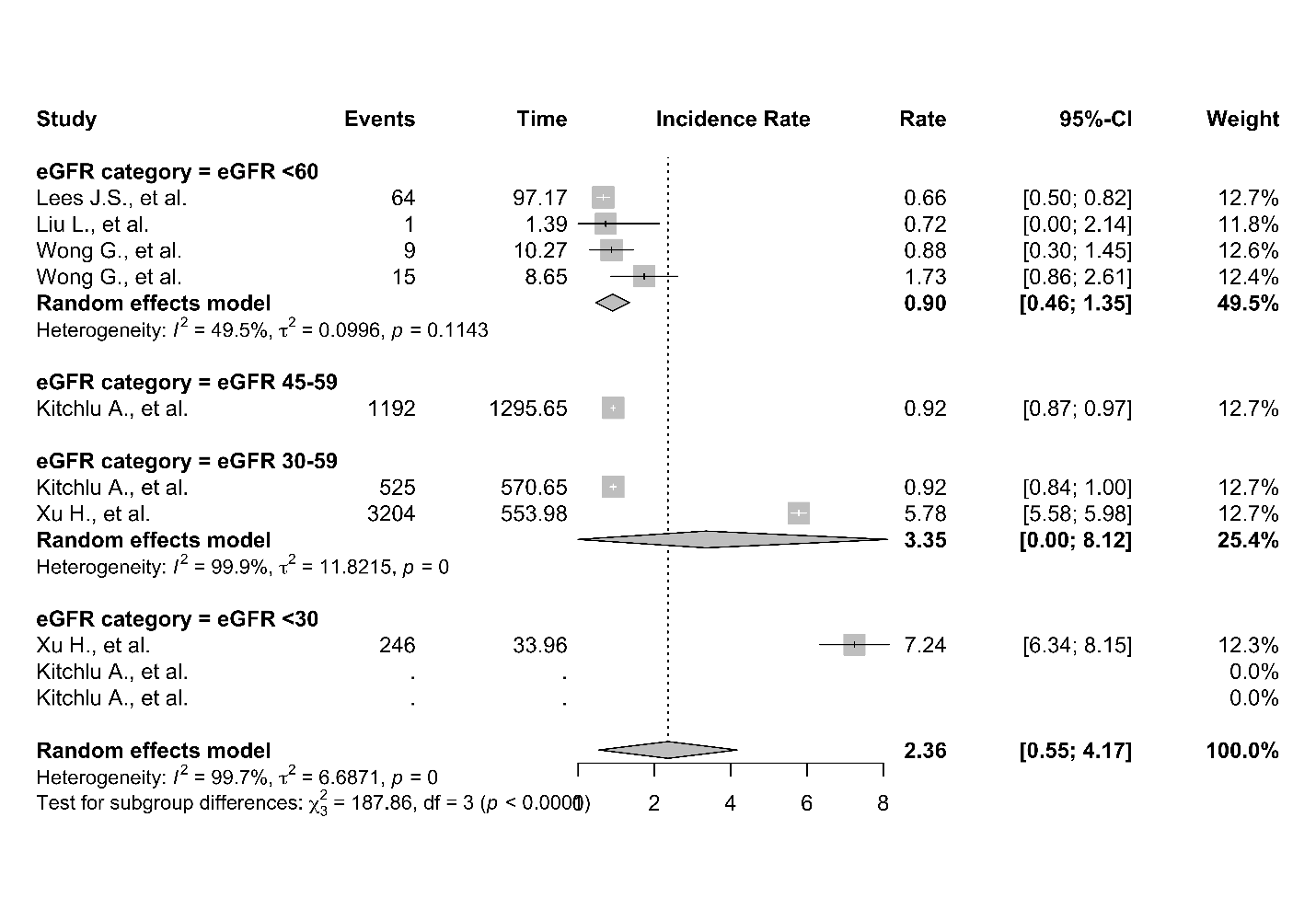


### **Supplementary Figures S7**

Urothelial cancer incidence in people with eGFR <60mL/min/1.73m^2^


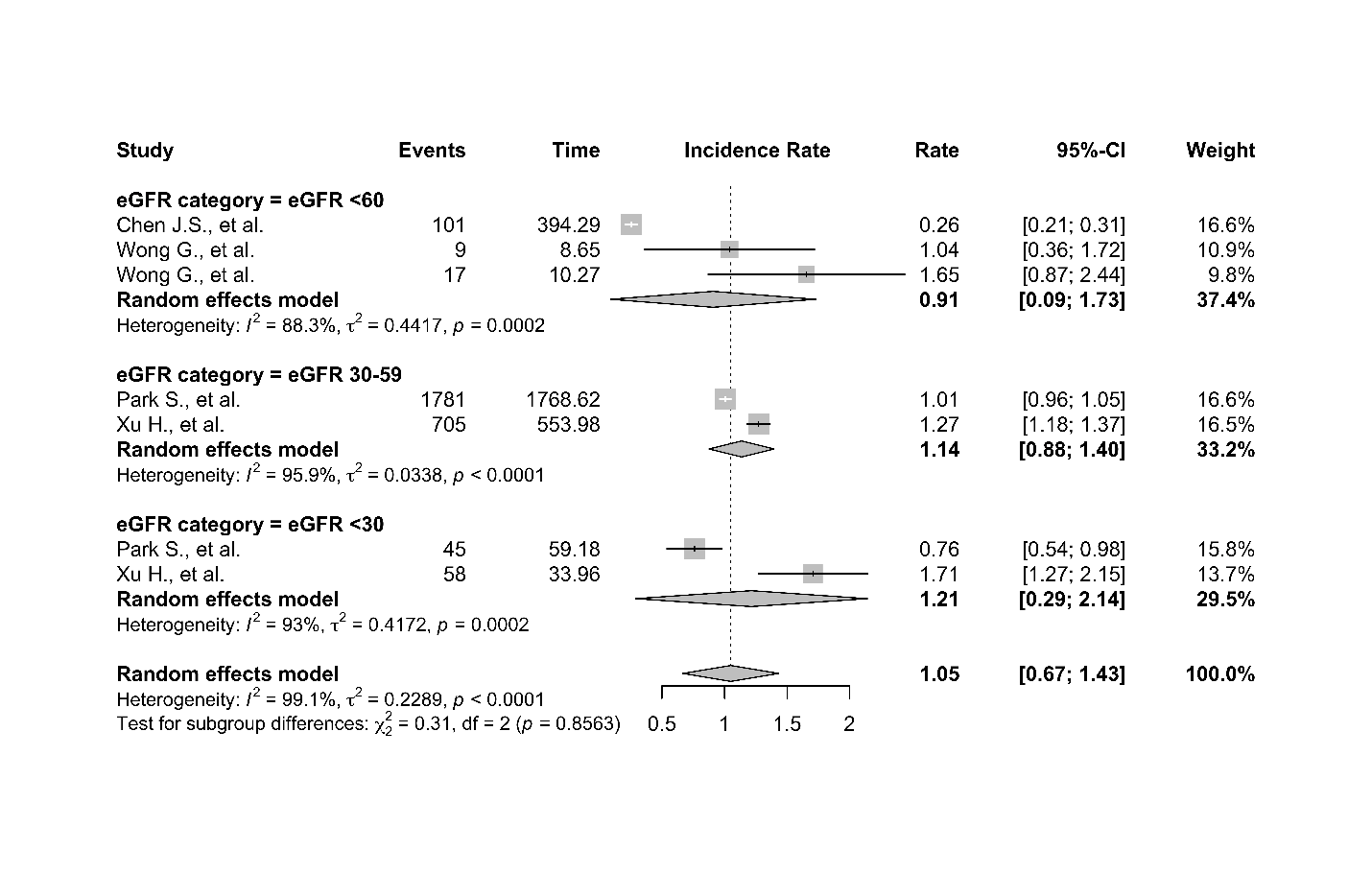


### **Supplementary Figures S8**

Breast cancer incidence in people with eGFR <60mL/min/1.73m^2^


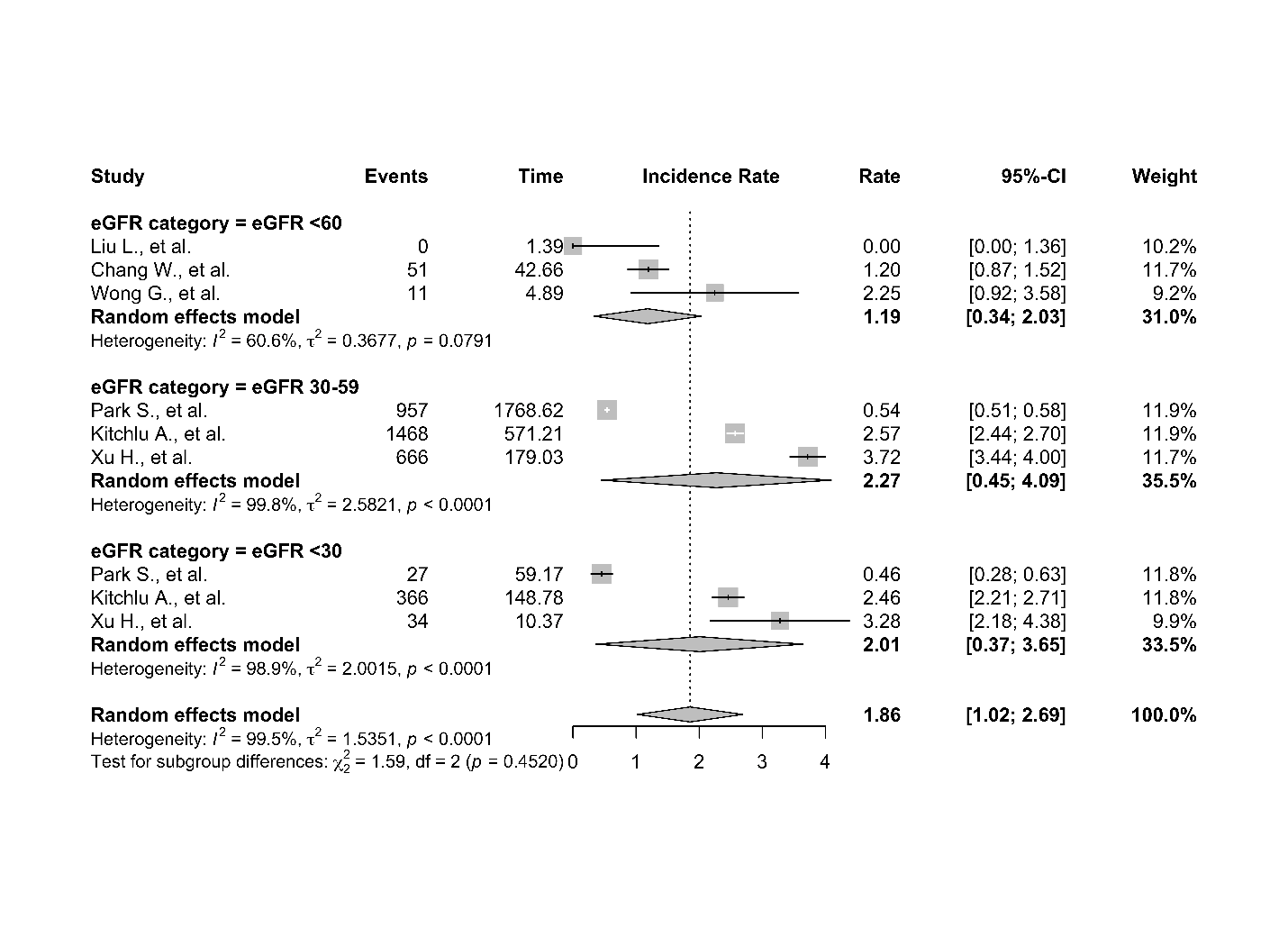


### **Supplementary Figures S9**

Prostate cancer incidence in people with eGFR <60mL/min/1.73m^2^


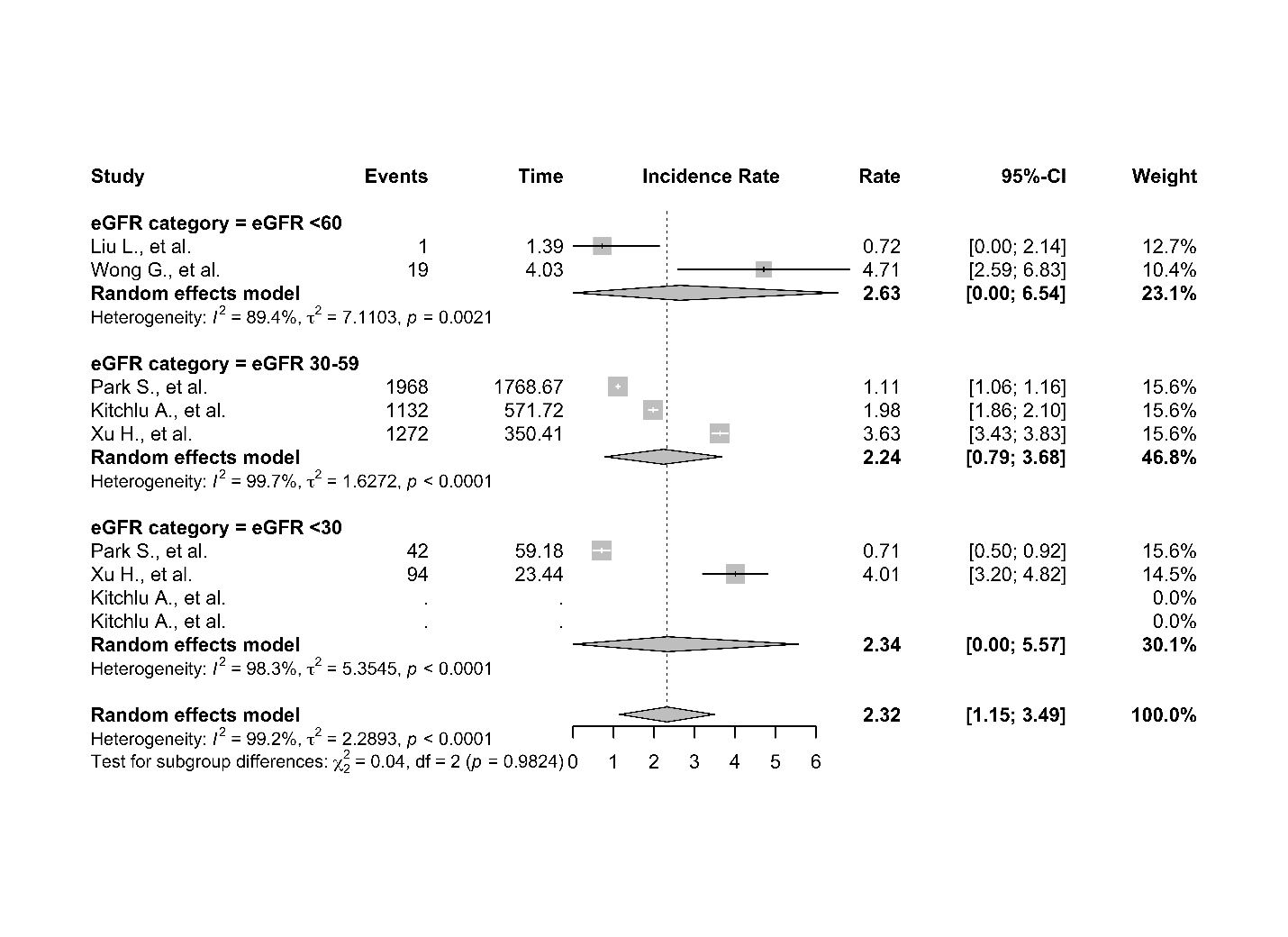


### **Supplementary Figures S10**

Cervical cancer incidence in people with eGFR <60mL/min/1.73m^2^


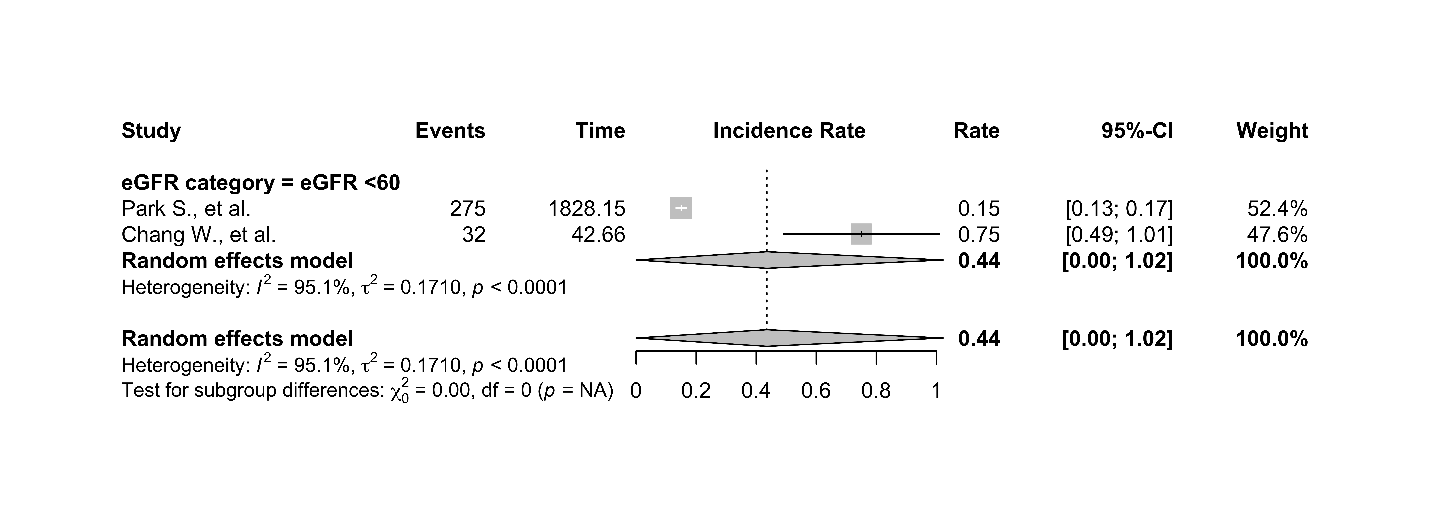


### **Supplementary Figures S11**

Funnel plot and reported p values for Egger’s and Begg’s tests for incidence rate ratios for people with a) eGFR ≥60 mL/min/1.73m^2^ and <60mL/min/1.73m^2^ b) eGFR ≥90 mL/min/1.73m^2^ and <60 mL/min/1.73m^2^ c) eGFR 60-89 mL/min/1.73m^2^ and <60 mL/min/1.73m^2^

a) Eggers’ test p = 0.336, Begg’s test p = 0.186


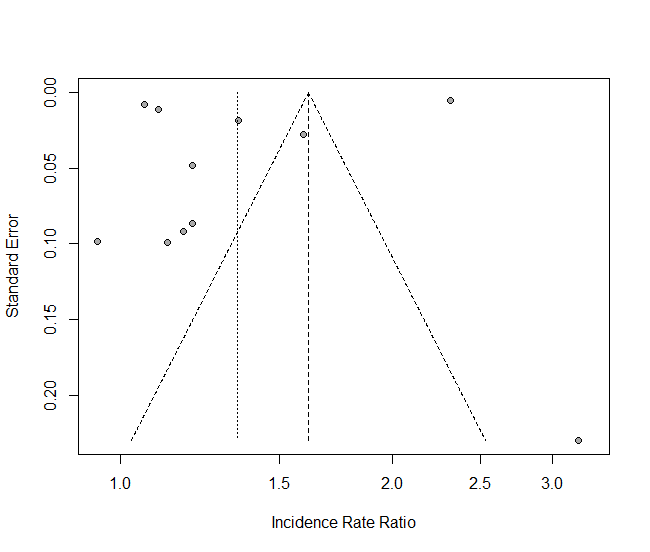


b) Eggers’ test p = 0.196, Begg’s test p = 1.000


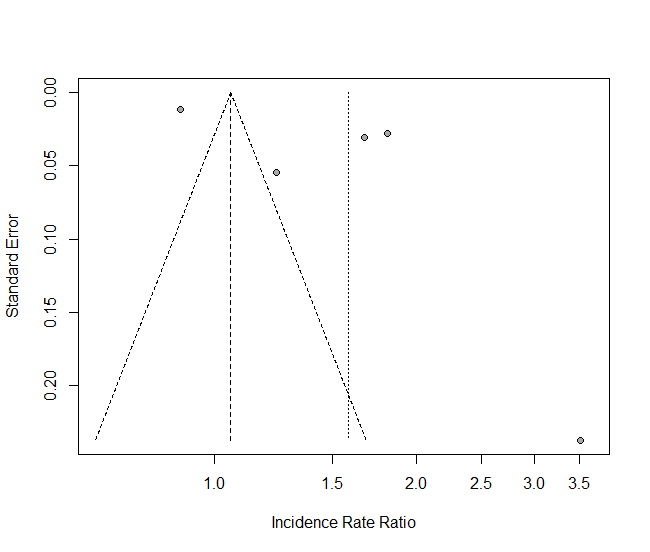


c) Eggers’ test p = 0.051, Begg’s test p = 0.091


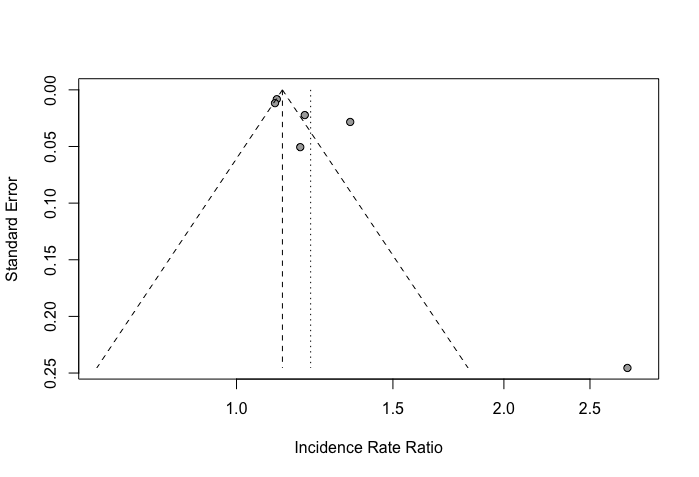


### **Supplementary Tables S1**

Newcastle-Ottawa Scale chart

| **Study** | **Selection** | | | | **Comparability** | **Outcome** | | | **Total** | **Overall Quality** |
| --- | --- | --- | --- | --- | --- | --- | --- | --- | --- | --- |
|  | **Representativeness of exposed cohorts** | **Selection non exposed cohort** | **Ascertainment of exposure** | **Demonstration that outcome of interest was not present at the start of the study** | **Comparability** | **Assessment of outcome** | **Was follow up long enough** | **Adequacy of follow up cohorts** |  |  |
| Chang W., et al.^43^ | * | * | * | * | ** | * | * | * | 9 | High |
| Chen J.-S., et al.^35^ | * | * | * | * | ** | * | * |  | 8 | High |
| Choi T., et al.^44^ | * | * | * | * | ** | * | * | * | 9 | High |
| Christensson A., et al.^45^ | * | * | * | * | ** | * | * |  | 8 | High |
| Chuang Y.-H., et al.^36^ |  | * | * | * | ** | * | * |  | 7 | High |
| Engel, SS., et al.^46^ |  | * | * |  | ** | * | * |  | 6 | Moderate |
| Er K.-C., et al.^52^ | * | * | * | * | ** | * | * | * | 9 | High |
| Hoang T., et al.^27^ |  | * | * | * | ** | * | * |  | 7 | High |
| Kitchlu A., et al.^30^ | * | * | * | * | ** | * | * | * | 9 | High |
| Lees J.S., et al.^12^ | * | * | * | * | ** | * | * | * | 9 | High |
| Liu L., et al.^47^ | * | * | * | * | ** |  | * | * | 8 | High |
| Lowrance W.T., et al.^29^ | * | * | * | * | ** | * | * | * | 9 | High |
| Miyamoto Y., et al.^48^ |  | * | * | * | ** | * | * | * | 8 | High |
| Mok Y., et al.^15^ |  | * | * | * | ** | * | * | * | 8 | High |
| Oh H.J., et al.^53^ | * | * | * | * | * | * | * |  | 7 | High |
| Oh H.J., et al.^49^ |  | * | * | * | ** | * | * | * | 8 | High |
| Park J., et al.^31^ | * | * | * | * | ** | * | * | * | 9 | High |
| Park S., et al.^26^ | * | * | * | * | ** | * | * | * | 9 | High |
| Sung F.-C., et al.^2^ |  | * | * | * | ** | * | * |  | 7 | High |
| Tendulkar K.K., et al.^28^ | * | * | * | * | ** | * | * |  | 8 | High |
| Tu H., et al.^54^ | * | * | * | * | ** | * | * | * | 9 | High |
| Wang C.-C., et al.^50^ | * | * | * | * | ** | * | * |  | 8 | High |
| Wong G., et al.^9^ | * | * | * | * | ** | * | * | * | 9 | High |
| Wong G., et al.^32^ |  | * | * | * | ** | * | * | * | 8 | High |
| Wu M.-Y., et al.^51^ | * | * | * | * | ** | * | * | * | 9 | High |
| Xu H., et al.^13^ | * | * | * | * | ** | * | * | * | 9 | High |
| Yu T.-Y., et al.^55^ |  | * | * | * | ** | * | * | * | 8 | High |

### **Supplementary Tables S2**

Baseline characteristics (including references)

| Authors | Year | Randomised Y/N | Study design | Country | Start date | End date | Sample size (n) | Cancer site(s) | CKD definition | eGFR categories | Proteinuria analysis |
| --- | --- | --- | --- | --- | --- | --- | --- | --- | --- | --- | --- |
| *Cancer incidence rates reported* | | | | | | | | | | | |
| Chang W., et al.^1^ | 2018 | N | Retrospective | Taiwan | 01/01/1996 | 31/12/2015 | 881430 | Gynae and Breast ca | ICD-9-CM 58 and 40 | >/<60 | x |
| Chen J.-S., et al.^2^ | 2016 | N | Retrospective | Taiwan | 01/01/1997 | 31/12/2011 | 1000000 | Upper tract urothelial carcinoma | Modification of Diet in Renal Disease equation | >/<60 | x |
| Choi T., et al.^3^ | 2022 | N | Retrospective | South Korea | 2009 | 31/12/2017 | 10505818 | Multiple myeloma | Modification of Diet in Renal Disease equation | >/<60, >120,119-90,89-60,59-30,<30 | Dipstick |
| Christensson A., et al.^4^ | 2013 | N | Prospective | Sweden | 1974 | 2006 | 33346 | All | Chronic Kidney Disease Epidemiology Collaboration (CKD-EPI) creatinine equation 2009 | >/<60 | x |
| Chuang Y.-H., et al.^5^ | 2021 | N | Retrospective | Taiwan | 2000 | 31/12/2015 | 471669 | Urothelial carcinoma | Chronic Kidney Disease Epidemiology Collaboration (CKD-EPI) creatinine equation 2009 | >90,89-60,59-45,<45 | Dipstick |
| Engel, SS., et al.^6^ | 2017 | Y | Prospective | International | 01/12/2008 | 01/07/2012 | 14671 | all cancer (other than non-melanoma skin cancers) | Not reported | >/<60 | x |
| Kitchlu A., et al.^7^ | 2022 | N | Retrospective | Canada | 01-Apr-07 | 31-Dec-17 | 6,246,941 | all | Chronic Kidney Disease Epidemiology Collaboration (CKD-EPI) creatinine equation 2009 | >60,59-45,44-30,30-15,<15 | x |
| Lees J.S., et al.^8^ | 2021 | N | Prospective | UK | 2007/2010 | 2020/2017 | 502493 | all cancer (other than non-melanoma skin cancers) | Chronic Kidney Disease Epidemiology Collaboration (CKD-EPI) creatinine equation 2009 | >90,89-60,<60 | uACR |
| Liu L., et al.^9^ | 2020 | N | Prospective | China | 2011 | 2015 | 17 708 | all cancer except minor skin cancer | Chronic Kidney Disease Epidemiology Collaboration (CKD-EPI) creatinine equation 2009 | >90,89-60,<60 | x |
| Lowrance W.T., et al.^10^ | 2014 | N | Retrospective | USA | 01/01/2000 | 01/12/2008 | 1,190,538 | renal and all except minor skin cancer | Chronic Kidney Disease Epidemiology Collaboration (CKD-EPI) creatinine equation 2009 | >120,119-90,89-60,59-30,<30 | x |
| Miyamoto Y., et al.^11^ | 2022 | N | Prospective | Japan | 1998 | 31/12/2013 | 24593 | all except minor skin cancer | modified IDMS–MDRD Study equation and the new Japanese equation | >90,89-60,59-45,<45 | Dipstick |
| Oh H.J., et al.^12^ | 2020 | N | Retrospective | South Korea | 2002 | 31/12/2013 | 514795 | colorectal ca | ICD codes: 'N18','N19' ,'I12', 'I13' ,'E10.2', 'E11.2', 'E13.2' and 'E14.2' | >/<60 | x |
| Park J., et al.^13^ | 2021 | N | Retrospective | South Korea | 2009 | 31/12/2016 | 10505818 | Kidney Ca | Modification of Diet in Renal Disease equation | >120,119-90,89-60,59-30,<30 | Dipstick |
| Park S., et al.^14^ | 2019 | N | Retrospective | South Korea | 2009 | 2016 | 18,936,885 | all cancer | Modification of Diet in Renal Disease equation | >/<60, >120,119-90,89-60,59-30,<30 | x |
| Sung F.-C., et al.^15^ | 2022 | N | Retrospective | Taiwan | 1999 | 2016 | 4578976 | HCC | Not reported | >/<60 | x |
| Wang C.-C., et al.^16^ | 2017 | N | Retrospective | Taiwan | 01/01/2000 | 2013 |  | NMSC | Chronic Kidney Disease Epidemiology Collaboration (CKD-EPI) creatinine equation 2009 | >/<60, <15 | x |
| Wong G., et al.^17^ | 2009 | N | Retrospective | Australia | 1993 | 2004 | 3448 | all except minor skin cancer | Modification of Diet in Renal Disease equation | >/<60 | x |
| Wong G., et al.^18^ | 2012 | Y | Prospective | International | 01/06/2001 | 01/06/2006 | 11140 | colorectal cancer, lung, prostate, urinary tract, breast and skin cancers | Modification of Diet in Renal Disease equation | >/<60 | x |
| Wu M.-Y., et al.^19^ | 2013 | N | Retrospective | Taiwan | 01/01/2004 | 31/12/2006 | 96843 | Colorectal cancer | ICD-9-CM codes | >/<60 | x |
| Xu H., et al.^20^ | 2019 | N | Prospective | Sweden | 01/01/2006 | 31/12/2012 | 1375156 | all cancer | Chronic Kidney Disease Epidemiology Collaboration (CKD-EPI) creatinine equation 2009 | >120,119-90,89-60,59-30,<30 | x |
| *Do not report stratified incidence rates* | | | | | | | | | | | |
| Er K.-C., et al.^21^ | 2016 | N | Retrospective | Taiwan | 01/01/2005 | 31/12/2013 | 985219 | Pancreatic ca | renal insufficiency | >/<60 | x |
| Oh H.J., et al.^22^ | 2018 | N | Retrospective | South Korea | 2002 | 2013 | 1025340 | Digestive ca | ICD codes: 'N18','N19' ,'I12', 'I13' ,'E10.2', 'E11.2', 'E13.2' and 'E14.2' | >/<60 | x |
| Tu H., et al.^23^ | 2018 | N | Prospective | Taiwan | 1996 | 31/12/2008 | 405878 | all cancer | National Kidney Foundation criteria | >90,89-60, <60 | x |
| Yu T.-Y., et al.^24^ | 2014 | N | Prospective | Taiwan | 01/07/1996 | 01/06/2003 |  | Hepatobiliary cancer, colorectal cancer and lung cancer | Chronic Kidney Disease Epidemiology Collaboration (CKD-EPI) creatinine equation 2009 | >/<60 | x |
| *Not included due to overlapping follow-up periods and geographical location* | | | | | | | | | | | |
| Hoang T., et al.^25^ | 2020 | N | Prospective | South Korea | 01/10/2007 | 31/12/2016 | 13644 | all | Modification of Diet in Renal Disease equation | >90,89-60,<60 | x |
| Mok Y., et al.^26^ | 2017 | N | Prospective | South Korea | 01/01/1996 | 31/12/2012 | 430920 | any cancer | Chronic Kidney Disease Epidemiology Collaboration (CKD-EPI) creatinine equation 2009 | >90,89-60,59-45,<45 | Dipstick |
| Tendulkar K.K., et al.^27^ | 2022 | N | Retrospective | USA | 01/01/2001 | 1/1/2001 | 1/12/2020 | all cancer (other than non-melanoma skin cancers) | Modification of Diet in Renal Disease equation | >60,59-45,44-30,<30 | x |

### **References**

1. Chang WH, Horng HC, Yeh CC, et al. Risks of female genital tract related cancers (gynecological cancers) or breast cancer in women with and without chronic kidney disease: A population-based cohort study in Taiwan. *Medicine (Baltimore)*. 2018;97(12):e0157. doi:10.1097/MD.0000000000010157
2. Chen JS, Lu CL, Huang LC, Shen CH, Chen SCC. Chronic Kidney Disease is Associated With Upper Tract Urothelial Carcinoma: A Nationwide Population-Based Cohort Study in Taiwan. *Medicine (Baltimore)*. 2016;95(14):e3255. doi:10.1097/MD.0000000000003255
3. Choi T, Ahn W, Shin DW, Han K, Kim D, Chun S. Association between Kidney Function, Proteinuria and the Risk of Multiple Myeloma: A Population-Based Retrospective Cohort Study in South Korea. *Cancer Res Treat*. 2021;54(3):926-936. doi:10.4143/crt.2021.951
4. Christensson A SC Sjoberg DD, Cronin AM, O’Brien MF, Lowrance W, Nilsson PM, Vickers AJ, Russo P, Lilja H. Association of cancer with moderately impaired renal function at baseline in a large, representative, population-based cohort followed for up to 30 years. doi:10.1002/ijc.28144
5. Chuang YH, Lin IF, Lao XQ, Lin C, Chan TC. The Association Between Renal Function Decline and the Incidence of Urothelial Carcinoma: A 16-year Retrospective Cohort Study in Taiwan. *Eur Urol Open Sci*. 2021;27:1-9. doi:10.1016/j.euros.2021.02.00457.
6. Engel S, Suryawanshi S, Stevens S, et al. Safety of sitagliptin in patients with type 2 diabetes and chronic kidney disease: outcomes from TECOS. *Diabetes Obes Metab*. 2017;19(11):1587‐1593. doi:10.1111/dom.12983
7. Kitchlu A, Reid J, Jeyakumar N, et al. Cancer Risk and Mortality in Patients With Kidney Disease: A Population-Based Cohort Study. *Am J Kidney Dis*. 2022;80(4):436-448.e1. doi:10.1053/j.ajkd.2022.02.020
8. Lees JS, Ho F, Parra-Soto S, et al. Kidney function and cancer risk: An analysis using creatinine and cystatin C in a cohort study. *eClinicalMedicine*. 2021;38. doi:10.1016/j.eclinm.2021.101030
9. Liu L, Zhu M, Meng Q, et al. Association between kidney function and the risk of cancer: Results from the China Health and Retirement longitudinal study (CHARLS). *J Cancer*. 2020;11(21):6429-6436. doi:10.7150/jca.47175
10. Lowrance WT, Ordoñez J, Udaltsova N, Russo P, Go AS. CKD and the Risk of Incident Cancer. *J Am Soc Nephrol*. 2014;25(10):2327. doi:10.1681/ASN.2013060604
11. Miyamoto Y, Katagiri R, Yamaji T, et al. Association of chronic kidney disease with total and site-specific cancer incidence in participants of the Japan Public Health Center-based Prospective Study. *Nephrol Dial Transplant*. 2023;38(6):1487-1496. doi:10.1093/ndt/gfac288
12. Oh HJ, Lee HA, Moon CM, Ryu DR. The Combined Impact of Chronic Kidney Disease and Diabetes on the Risk of Colorectal Cancer Depends on Sex: A Nationwide Population-Based Study. *Yonsei Med J*. 2020;61(6):506-514. doi:10.3349/ymj.2020.61.6.506
13. Park J., Shin D.W., Han K., Kim D., Chun S., Jang H.R. Associations Between Kidney Function, Proteinuria, and the Risk of Kidney Cancer: A Nationwide Cohort Study Involving 10 Million Participants. *Am J Epidemiol*. 2021;190(10):2042-2052. doi:10.1093/aje/kwab140
14. Park S, Lee S, Kim Y, et al. Risk of cancer in pre-dialysis chronic kidney disease: A nationwide population-based study with a matched control group. *Kidney Res Clin Pract*. 2019;38(1):60-70. doi:10.23876/j.krcp.18.0131
15. Sung H, Ferlay J, Siegel RL, et al. Global Cancer Statistics 2020: GLOBOCAN Estimates of Incidence and Mortality Worldwide for 36 Cancers in 185 Countries. *CA Cancer J Clin*. 2021;71(3):209-249. doi:10.3322/caac.21660
16. Wang C.-C., Tang C.-H., Huang S.-Y., Huang K.-C., Sue Y.-M. Risk of non-melanoma skin cancer in patients with chronic kidney disease and its relationship to uraemic pruritus. *Acta Derm Venereol*. 2017;97(10):1230-1234. doi:10.2340/00015555-2762
17. Wong G, Hayen A, Chapman JR, et al. Association of CKD and Cancer Risk in Older People. *J Am Soc Nephrol*. 2009;20(6):1341. doi:10.1681/ASN.2008090998
18. Wong G, Zoungas S, Lo S, et al. The risk of cancer in people with diabetes and chronic kidney disease. *Nephrol Dial Transplant*. 2012;27(8):3337‐3344. doi:10.1093/ndt/gfs022
19. Wu MY, Chang TC, Chao TY, Huang MT, Lin HW. Risk of Colorectal Cancer in Chronic Kidney Disease: A Matched Cohort Study Based on Administrative Data. *Ann Surg Oncol*. 2013;20(12):3885-3891. doi:10.1245/s10434-013-3065-8
20. Xu H, Matsushita K, Su G, et al. Estimated Glomerular Filtration Rate and the Risk of Cancer. *Clin J Am Soc Nephrol*. 2019;14(4):530. doi:10.2215/CJN.10820918
21. Er KC, Hsu CY, Lee YK, Huang MY, Su YC. Effect of glycemic control on the risk of pancreatic cancer: A nationwide cohort study. *Medicine (Baltimore)*. 2016;95(24):e3921. doi:10.1097/MD.0000000000003921
22. Oh H.J., Lee H.A., Moon C.M., Ryu D.-R. Incidence risk of various types of digestive cancers in patients with pre-dialytic chronic kidney disease: A nationwide population-based cohort study. *PLoS ONE*. 2018;13(11):e0207756. doi:10.1371/journal.pone.0207756
23. Tu H, Wen CP, Tsai SP, et al. Cancer risk associated with chronic diseases and disease markers: prospective cohort study. *BMJ*. 2018;360:k134. doi:10.1136/bmj.k134
24. Yu TY, Li HY, Jiang YD, et al. Serum Vascular Adhesion Protein-1 Level Predicts Risk of Incident Cancers in Subjects with Type II Diabetes. *Cancer Epidemiol Biomarkers Prev*. 2014;23(7):1366-1373. doi:10.1158/1055-9965.EPI-14-0023
25. Hoang T, Lee J, Kim J. Comorbidity Risk Score in Association with Cancer Incidence: Results from a Cancer Screenee Cohort. *Cancers*. 2020;12(7):1834. doi:10.3390/cancers1207183
26. Mok Y, Matsushita K, Ballew SH, et al. Kidney Function, Proteinuria, and Cancer Incidence: The Korean Heart Study. *Am J Kidney Dis*. 2017;70(4):512-521. doi:10.1053/j.ajkd.2017.03.018
27. Tendulkar KK, Cope B, Dong J, Plumb TJ, Campbell WS, Ganti AK. Risk of malignancy in patients with chronic kidney disease. *PLOS ONE*. 2022;17(8):e0272910. doi:10.1371/journal.pone.0272910
